# Exploring the visual system of the black grouse (*Lyrurus tetrix*): combining experimental and molecular approaches to inform strategies for reducing collisions

**DOI:** 10.1242/jeb.250727

**Published:** 2026-04-24

**Authors:** Simon Potier, Jean-Marc Lassance, Constance Blary, Justine Coulombier, Sandrine Berthillot, Jérôme Cavailhes, Charleyne Buisson, Virginie M. Dos Santos, Christine Andraud, Marjorie A. Liénard

**Affiliations:** ^1^ Simon Potier, Expert Scientifique, 27220 Foucrainville, France; ^2^ GIGA Institute, University of Liège, 4000 Liège, Belgium; ^3^ CEFE, Univ Montpellier, CNRS, EPHE, IRD, 34090 Montpellier, France; ^4^ Parc national de la Vanoise, 73000 Chambéry, France; ^5^ Association Observatoire des Galliformes de Montagne, 74320 Sevrier, France; ^6^ Centre de Recherche sur la Conservation des Collections, CNRS, Muséum National d'Histoire Naturelle, 75005 Paris, France; ^7^ Laboratory of Molecular Biology of Sensory Systems, GIGA Institute, University of Liège, 4000 Liège, Belgium

**Keywords:** Visual field, Contrast vision, Spectral sensitivity, Opsin, Bird collision

## Abstract

Understanding the visual systems of birds can inform conservation efforts and mitigate the impact of collisions with human-made structures. Here, we investigated the visual abilities of the black grouse, *Lyrurus tetrix* (Galliformes: Phasianidae), a European mountain bird highly vulnerable to collisions with aerial infrastructures. We show that black grouse have wide monocular lateral visual fields, extensive binocular overlap and a minimal blind area behind the head, altogether indicating the ability to detect aerial objects from different fields of view. The spatial resolution and contrast sensitivity of the black grouse are in the low avian range but align with the ecology of a prey species and allow the establishment of the limits of detection of an object size and contrast under variable environmental conditions. To characterize the range of wavelengths perceived, the four cone visual pigments (SWS1, SWS2, Rh2 and LW) were reconstituted and characterized functionally, showing detection of light across a spectral range spanning the visible spectrum up to 650 nm and with limited sensitivity below 400 nm. Combined with spectral and achromatic modelling analyses, our results inform on the limits of detection of aerial objects and the perception of existing visual markers that are currently employed to mitigate black grouse collisions.

## Teaser

**Highlighted Article:** Investigation of how black grouse perceive their environment suggests how vision-informed marker design may improve detection of aerial structures and reduce collision risk.

## INTRODUCTION

Collisions are a recognized major source of bird mortality worldwide (Rosenberg et al., 2019; Loss et al., 2014), especially with glass or other reflective building surfaces (Machtans et al., 2013), wind turbines (Marques et al., 2014; Drewitt and Langston, 2008), power lines (Bernardino et al., 2018) and aerial cables that intrude into the open airspace. With the rise of ski resorts in alpine regions, the development of road networks, ground and aerial infrastructures and cable transport systems (e.g. catex, cable cars, surface lifts) has substantially reshaped landscapes over the last 50 years, notably increasing habitat fragmentation and reducing natural wildlife habitats and refuges for mountain avifauna, in particular for ground-nesting birds (Watson and Moss, 2004; Segelbacher et al., 2008; Bech et al., 2013; Matysek et al., 2021). Ski resort areas have also been associated with high collision rates, especially for Galliformes (Miquet, 1990; Bevanger, 1998; Buffet and Dumont-Dayot, 2013).

Black grouse, *Lyrurus tetrix* (Phasianidae, previously *Tetrao tetrix*) are a highly sedentary ground-nesting gallinaceous bird that forages for terrestrial invertebrates, berries, plant shoots or leaves (Hambálková et al., 2024). Black grouse are sensitive to habitat change and human disturbances and are commonly exposed to a range of collision hazards. Documented effects include deer fences in Scotland populations (Baines and Summers, 1997), power lines in Scandinavia (Bevanger, 1995) and ski lift cables in Alpine regions (Miquet, 1990; Buffet and Dumont-Dayot, 2013). When comparing black grouse population abundance in natural habitats versus ski resorts in the south-western Swiss alps, Patthey et al. (2008) quantified that habitat topology and ski lift density were associated with a 15% reduction in local black grouse abundance but without linking it to collisions per se (Patthey et al., 2008). In the French alps, where black grouse populations are smaller and more isolated than in Scandinavian regions, around 400 deaths of black grouse have been recorded near aerial infrastructures since 1964, which represents ca. 70% of the total bird death counts over that period (Corona and Dos Santos, 2023; https://www.observatoire-galliformes-montagne.com/Tetras-lyre.html). According to earlier estimates of the French Alpine black grouse population in 2010 (https://www.observatoire-galliformes-montagne.com/Tetras-lyre.html), collisions caused mortality accounting for 2.5% of the total population. This number is probably conservative as it excluded individuals that collide in flight but might die outside search areas, and as the probability of detecting carcasses is very low (Bech et al., 2012).

**Fig. 1.**
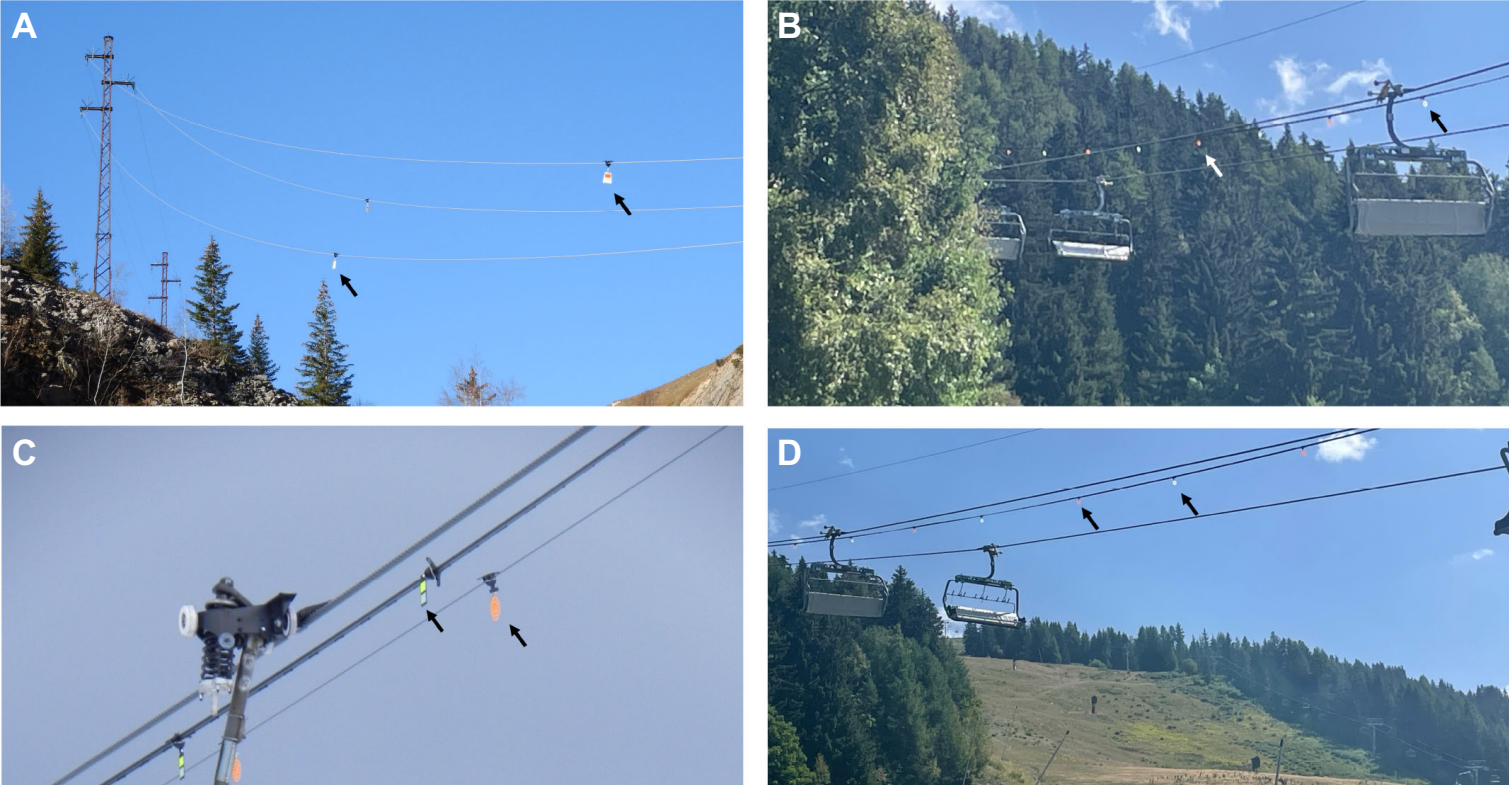
**Aerial cables and ski lifts equipped with visual markers in the French Alps.** Visual markers are indicated with arrows (see also Table S3 and Fig. 7 for alternative designs). (A) Example of *black Firefly* markers (15×9 cm) on aerial electrical cables. (B–D) Examples of *Crocfast* and *Birdmarks* markers (13.5 cm in diameter) seen against (B) a green background, (C) a cloudy background and (D) a clear sky. Photo credit: V.P.N. (A,C) and J.-M.L. (B,D).

For more than 20 years, numerous ski resorts have equipped ski lifts with visual markers, small plastic or metallic devices (Fig. 1; Table S1) designed to increase visibility through size, movement in the wind, reflective properties and contrasting colour patterns (Dos Santos et al., 2021). However, the effectiveness of these markers can differ among target species and environmental conditions (Ferrer et al., 2020), and it remains unclear whether black grouse perceive these hazard markers or obstacles in their flight paths. To assess current solutions and possibly guide the development and efficient placement of visual markers, knowledge of avian species-specific vision, ecology and behaviour should necessarily be considered (Martin, 2011a, 2022; Martin and Banks, 2023).

Vision is the primary sensory modality for most avian species, collecting accurate and instantaneous environmental information from different directions and over varying distances (Land and Nilsson, 2012). Avian visual systems influence object detection for various tasks, and are shaped by natural history and ecology such as diel activity patterns, navigation, foraging strategies or predator–prey interactions (Odeen and Håstad, 2010; Wu et al., 2016; Outomuro et al., 2017; Hauser and Chang, 2017; Cooney et al., 2019; Caves et al., 2024; Blary et al., 2024).

While detection of an object is influenced by its inherent achromatic and chromatic properties, bird flight behaviour and the environmental light conditions, together with the bird's visual characteristics including its visual field, spatial resolution as well as both contrast and spectral sensitivity appear to be important factors (Martin, 2022). The visual field is the volume of space around the head from which visual input is gathered at any given time (Martin, 2017a). It therefore determines whether an artificial structure or object falls within a blind area or within a bird's field of view. In the latter case, the binocular region allows estimation of time before contact (Martin, 2009, 2017b; Martin et al., 2012).

An object must also reach a minimum size at a distance threshold to be detected prior to collision (Martin, 2022), which can be informed by determining spatial resolution. Spatial resolution is the ability to resolve two points as separate, and is a measure also used to quantify how well birds can distinguish fine details, defining their visual acuity (Land and Nilsson, 2012). Target detection further requires the ability to discern contrasts between an object and the background, or patterns within an object, and is defined by the visual system's contrast sensitivity. Finally, the spectral sensitivity of the avian eye is determined by different photoreceptors, which shape how birds detect wavelengths of light and perceive colours (Potier et al., 2020; Kelber, 2019; Stavenga and Wilts, 2014; Wu et al., 2016). Most avian visual systems have four single cone types, each containing one opsin that, when bound to 11-cis-retinal, forms a visual pigment with a distinct maximal wavelength sensitivity (λ_max_) spanning the following ranges across taxa: SWS1 360–420 nm (ultraviolet sensitive <385 nm, violet-sensitive >385 nm), SWS2 430–460 nm, Rh2 470–510 nm, LW 530–560 nm (Carvalho et al., 2007, reviewed in Hauser et al., 2014; Hagen et al., 2023). Galliformes investigated to date via physiological and molecular studies exhibit violet-sensitive SWS1 cones (Ödeen and Håstad, 2003; Hart and Hunt, 2007). Bird cone opsins typically mediate a tetrachromatic colour vision system, in combination with cone-specific oil-droplet filters, resulting in substantial interspecific variation in visual capabilities (Kelber, 2019). Studying species-specific visual characteristics can therefore inform the limits of object detection in both natural and human-made environments.

Despite longstanding conservation interest in black grouse, many aspects of their visual capabilities remain unknown. Here, we used a combination of reflex-like behaviours, ophthalmoscopy and molecular biology to assess their visual fields, spatial resolution, contrast sensitivity and wavelength absorbance of cone visual pigments. We then measured the reflectance properties of anti-collision visual markers placed on ski lifts and discuss the black grouse visual system characteristics in the context of marker detection, as well as the risk of flight collision under variable visual environmental conditions.

## MATERIALS AND METHODS

### Birds and study location

Two 4-month-old male black grouse, *Lyrurus tetrix* (Linnaeus 1758), individuals belonging to the Frank Grosemans' breeding centre (Belgium) were used to study visual fields, spatial resolution and contrast sensitivity.

Ethical approval for the handling and restraint of birds was granted under the permit number APAFIS# 31775-31775-2021041311196892 v5. Permissions were granted from the owner of the birds used in the study.

### Visual fields

A non-invasive procedure was used to measure visual field characteristics in alert birds (Martin and Portugal, 2011). Each bird was held firmly for 20–30 min in a plastic restraining tube of the appropriate size to avoid any movement, with its legs cushioned by foam rubber and gently immobilized by taping them together with surgical tape (Micropore 1530/1B). The head was held in a natural position and placed at the centre of a visual field apparatus, a device that permits the eyes to be examined from known positions around the head. The bill was immobilized in a specifically manufactured steel and aluminium bill holder, and held with micropore tape, keeping the nostrils clear for normal breathing. The bill holder surfaces were coated with cured silicone sealant to provide a cushioned, non-slip surface. The bird's head and eyes were photographed (iPhone 13, Apple) while in the apparatus to determine the eye positions in the skull, the horizontal separation between the centre of both eyes, the eye-to-bill-tip distance and the bill length.

Visual field parameters were measured using an ophthalmoscopic reflex technique, with the perimeter's coordinate system following latitude–longitude conventions and the equator aligned to the median sagittal plane, which divides the head vertically and symmetrically into left and right hemispheres. This coordinate system is used throughout the presentation of the results. We first examined the eyes using an ophthalmoscope mounted on the perimeter arm with an accuracy of ±0.5 deg to measure the boundaries of the retina projection from the positions that the eyes spontaneously adopted when they were fully rotated forwards, i.e. converged for estimation of binocular area boundaries, and backwards, i.e. diverged for estimation of the blind area behind the head. The degree of eye movements and pecten projection were not measured to minimize restraint time.

We corrected our measurements to a hypothetical viewing point placed at infinity, based on the distance used in the visual field apparatus and the horizontal separation of the eyes (Martin, 1984). Using these corrected values, we constructed a topographical map of the visual field, including the lateral fields, binocular field, cyclopean field, i.e. the total field around the head comprising the combined monocular fields of both eyes, and blind areas above and behind the head. The limits of the visual field were assessed at 10 deg elevation intervals along an arc, enabling a complete estimation of the binocular field sector. At elevations where the bill holder blocked our view, binocular field width was interpolated as the mean of the binocular field widths immediately above and below these elevations (Martin and Portugal, 2011).

### Spatial resolution

Spatial resolution, or visual acuity, is the ability of the eye to resolve spatial detail when the contrast in the stimulus is high. Spatial resolution is often measured at high (day-time) light levels (Martin, 2017a). Spatial resolution can be estimated from retinal photoreceptor or ganglion cell density in the retina (Coimbra et al., 2012; Lisney et al., 2012), estimates that closely correlate with behavioural measures of visual acuity across vertebrates (Caves et al., 2024). In birds, especially diurnal species, there is often a strong positive correlation between visual acuity and eye size, particularly axial length (Kiltie, 2000; Caves et al., 2024), although this correlation does not necessarily apply to all birds, as ostriches despite having large eyes exhibit lower acuity than some smaller-eyed raptors (Boire et al., 2001; Caves et al., 2024). Axial length was obtained by measuring corneal diameter, offering the advantage of being non-destructive, while providing estimates of visual acuity that are comparable to those obtained from behavioural estimation (Potier et al., 2016).

Corneal diameter (CD) was measured from calibrated eye photographs with ImageJ 1.41 (Schneider et al., 2012) to obtain axial length (AL, in mm) using the formula for diurnal animals (Hall and Ross, 2007):
(1)

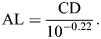
Spatial resolution (SR; in cycles per degree, abbreviated to cpd) was calculated following the function derived from Caves et al. (2024):
(2)

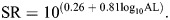


### Contrast sensitivity

#### Experimental set-up

We used an eye reflex-like behaviour setup to study the contrast sensitivity of each individual, following Wagner et al. (2022) and Blary et al. (2024). This approach yields peak contrast sensitivities comparable to those obtained with the operant conditioning method (Blary et al., 2024). The eye reflexes, or optocollic reflexes, are caused by involuntary compensatory mechanisms for image stabilization, i.e. the bird follows the moving object by eye to keep the image stationary on the retina (Wagner et al., 2022). Because most birds have limited eye movements between 2 and 18 deg (Martin, 2009; O'Rourke et al., 2010), they follow the object with large head movements. To estimate the optocollic reflex (OCR), birds were placed into a U-shaped box adapted to their size (60×40×44 cm; L×W×H), with a black wooden floor and ceiling, and clear polycarbonate walls (3 mm) (Fig. 3A). The wall allows the bird to see the stimulus without influencing visual ability (Blary et al., 2024). The ceiling consisted of two wooden boards spaced 5 cm apart: a perforated panel to allow air flow through and a smaller solid panel preventing the bird from seeing through the holes. All wooden surfaces were painted matte black (Liberty black Matt, GoodHome). A rear wooden door allowed the bird to be positioned inside the box. The box was placed at the centre of an octagon (radius 69 cm) formed by computer monitors (Acer KG1 Series, 28 inches, 4K 3840×2160 pixels, 60 Hz) in portrait orientation, with their centres aligned to the level of the bird's head. The stimulus covered 236×46 deg of the visual field (horizontal×vertical), sufficient to elicit an OCR (Schmid and Wildsoet, 1998; Blary et al., 2024).

The monitors and the bird were enclosed in an opaque black cloth tent (300×300×250 cm) with the monitor bases and floor also covered in black cloth to minimize external visual cues. The experimenter remained outside the tent throughout the experiment. A video camera (25 frames s^−1^; Sony DCR-SR55 HD) positioned above the monitors recorded the bird's behaviour and a smartphone (iPhone 13, Apple) connected to an external screen allowed continuous remote monitoring.

#### Stimuli

Stimuli consisted of vertical sinusoidal achromatic gratings of varying spatial frequencies and contrasts generated in Matlab (R2021) using Psychtoolbox (Brainard, 1997). Monitors were driven by MacBook Pro (Apple) via an 8-Port 4K/60Hz HDMI splitter (ST128HD20, StarTech). The angular velocity of the gratings was set to 20 deg s^−1^, matching values used in closely related species (Blary et al., 2024). To obtain the contrast sensitivity function (CSF) – including peak contrast sensitivity, its corresponding spatial frequency and the high cut-off frequency (i.e. the highest spatial frequency detected at a Michelson contrast of 1) – we used six spatial frequencies (0.47, 0.56, 0.85, 1.06, 1.27 and 1.59 cpd) and Michelson contrasts ranging from 0.012 to 0.99 (Table S2). Specifically, Michelson contrast (*C*_m_) is defined as:
(3)

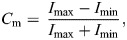
where *I*_min_ and *I*_max_ are the minimum and maximum luminance; values range from 0% (no contrast) to 100% (perfect black–white difference). For each contrast level, luminance differences between the darkest and lightest regions of the monitor centre were measured with a luxmeter (Hagner ScreenMaster, B. Hagner, Bålsta, Sweden) and expressed in candelas per square metre (cd m^−2^).

Each stimulus was displayed for 20 s (Schmid and Wildsoet, 1998; Shi and Stell, 2013; Wagner et al., 2022; Blary et al., 2024), with stimulus direction (clockwise or anticlockwise) varied in a pseudorandom order, limiting consecutive identical directions to three. Illuminance at the bird's head was 110 lx inside the U-shaped box and 321 lx outside, and stimulus brightness was 73 cd m^−2^.

#### Experimental procedure

Computer monitors were switched on 20 min before to the experiment to reach maximum brightness. Following the bird's placement in the device, a 10 min habituation period was observed before the experimental session began. The experimenter visually assessed the presence of the OCR in accordance with standard practice (Schmid and Wildsoet, 1998; Shi and Stell, 2013; Blary et al., 2024). Each session began with maximum contrast (Michelson contrast=0.99) to ensure that the bird was expressing an OCR detectable by the experimenter. Contrast was then varied stepwise as described in Blary et al. (2024), to identify the threshold at which birds no longer exhibited the reflex. Each trial combined a specific combination of spatial frequency and contrast and was separated by at least 5 s of an isoluminant homogeneous grey screen (longer if the bird did not look at the monitors). We applied the ‘four of five’ criteria: each combination was presented 5 times, and a response was considered reliable if the OCR occurred in at least 4 trials (Shi and Stell, 2013; Blary et al., 2024). The lowest contrast meeting this criterion was defined as the threshold contrast. Once this was determined, the experiment was repeated at the same angular velocity with a different spatial frequency. All session were video-recorded, allowing retrospective verification of OCR presence or absence.

### Opsin λ_max_ characterization via heterologous expression

#### Collection of eye tissues and extraction of RNA

The retina was dissected by a veterinarian from an injured adult female black grouse collected in Bourg-Saint-Maurice, France (ID: JB-2021-E001, POIA Birdski Project 2020-2023 Vanoise). The collected tissue was directly preserved in DNA/RNA reagent (Zymo Research) and stored at −20°C. Before RNA extraction, the tissue sample was removed from the stabilization solution, briefly dried and then ground to a fine powder in a mortar cooled with liquid nitrogen. We then proceeded with RNA extraction with a Monarch Total RNA miniprep kit (NEB) following the manufacturer's instructions. The integrity of the RNA was evaluated using a bioanalyser and the quantity assessed using a Qubit High Sensitivity RNA assay (Invitrogen).

#### cDNA libraries and RNA sequencing

High-quality RNA (500 μg) was used to prepare a DNA library following the protocol detailed in NEBNext Ultra II Directional RNA library prep kit for Illumina (NEB), including the following specifications: selection of mRNA, a selected average size of 500 base pairs (bp) for fragmentation, and the addition of Illumina adapters. The quality of the cDNA library was evaluated using a bioanalyser as well as by quantitative (q)PCR. Paired-end sequencing (2×150 bp) was performed on an Illumina NovaSeq6000 equipped with an S4 flowcell (NovaSeq Control Software 1.8.0/RTA v3.4.4, SciLifeLab, Stockholm, Sweden). In total, we obtained 197.8 million read pairs. The transcriptome sequencing data have been deposited in the European Nucleotide Archive (ENA) under the project accession PRJEB86944.

#### Pre-assembly filtering

Raw reads were first pre-processed by correcting and excluding aberrant reads using the k-mer-based read-error correction tool Rcorrector (Song and Florea, 2015). We next proceeded to adapter removal and quality trimming (quality-cutoff=20) using cutadapt version 4.2 (Martin, 2011b). During this step, we also removed read pairs with either read shorter than 36 bp. Next, we removed reads corresponding to rRNA sequences by mapping the reads against a custom database built from SILVA SSU and LSU datasets (release 138.1) (Quast et al., 2013) using bowtie2 (Langmead and Salzberg, 2012). Finally, reads corresponding to mitochondrial sequences were extracted using MITGARD (Nachtigall et al., 2021). The final set of assembly-ready reads contained 173.8 million read pairs.

#### *De novo* transcriptome assembly and annotation

*De novo* assembly of the pre-processed paired-end reads was performed using Trinity v2.14.0 (Garber et al., 2011) with default parameters. Completeness of the assembly was assessed with BUSCO v3.0.2 (Simão et al., 2015) using the Vertebrata and Aves datasets. We applied the pipeline implemented in Trinotate v3.2.1 (Bryant et al., 2017) to predict candidate protein coding regions within transcript sequences using TransDecoder v5.5.0 (https://github.com/TransDecoder/TransDecoder) and to generate a functional annotation of the transcriptome data. We retrieved full-length candidate opsin transcripts from the resulting annotation table (Dataset S1 in https://figshare.com/s/dc412ea22f2eca8ff869).

#### Opsin functional expression

Opsin-specific oligonucleotide primers (Table S3) encompassing two distinct restriction sites were designed to amplify the coding region of each identified visual opsin (SWS1, SWS2, Rh2, LW) by PCR using retinal cDNA synthesized using the GoScript protocol (Promega) as template. After verification by agarose gel electrophoresis, expected amplified product sizes were gel-purified using the Monarch DNA gel extraction kit (NEB), digested with restriction enzymes, subcloned into the linearized pcDNA5-FLAG-T2A-mruby2 expression vector (Liénard et al., 2021) and propagated into a Q5 strain of *Escherichia coli* bacteria (NEB). After incubation of individual bacterial colonies in Luria–Bertani (LB) culture medium containing ampicillin, DNA plasmids were purified, and each construct sequence was verified by Sanger sequencing. A stock of 480 µg for each final plasmid was prepared following the ZymoPURE II Plasmid MidiPrep kit protocol (Zymo).

#### Protein purification, absorbance measurement and UV-VIS analysis

Each plasmid was transfected into human embryonic kidney cells (HEK293T, ThermoFisher) following the PASHE procedure (Liénard et al., 2022). Briefly, 11-cis-retinal was added in solution in the dark (under red light) 6 h post-transfection. Cells were collected and the total membrane fraction solubilized using 1% *n*-dodecyl-d-maltoside detergent (DDM) 48 h after transfection, and the opsin fraction bound to FLAG resin and incubated overnight. The next day, the opsin–FLAG complex was purified and eluted using FLAG peptide, subsequently concentrated by centrifugation at 4000 rpm and 4°C for 50 min prior to ultraviolet-visible (UV-VIS) spectroscopy. The absorbance of each purified rhodopsin (opsin in complex with the chromophore) was measured in the dark from 1.5 μl aliquots using a NanoDrop 2000/2000c UV-VIS spectrophotometer (ThermoFisher). For each rhodopsin, we estimated λ_max_ by non-linear fitting of the absorbance data using a visual pigment template (Govardovskii et al., 2000). We performed 1000 bootstrap replicates to compute λ_max_ estimates and the associated confidence intervals (Liénard et al., 2022) in R v3.6.6. using the rsample (https://CRAN.R-project.org/package=rsample) and tidymodels (https://CRAN.R-project.org/package=tidymodels) packages.

### Modelling of spectral sensitivity in cone photoreceptors

To derive a theoretical effective eye spectral sensitivity in the black grouse, which depends on the visual pigment opsin λ_max_ and oil droplet filtering, we modelled the relationship between each cone opsin λ_max_ and a parsimonious microspectrophotometric oil droplet for Galliformes (Stavenga and Wilts, 2014). The mid-wavelengths of absorbance, i.e. where the droplet transmits 50% (or λ_mid_) were inferred for the C, Y and R oil droplet types as follows: For the C-type droplet expressed in SWS2, the established empirical relationship is approximated as C_mid_=0.82×λ_max_SWS2_+75, providing C-type λ_mid_ at 430–434 nm (Hart and Vorobyev, 2005); for Y- and R-type droplets expressed in Rh2 and LW cones, Y_mid_ 523 nm and R_mid_ 586 nm were applied (Hart and Vorobyev, 2005; Bowmaker et al., 1997; Hart and Hunt, 2007). Droplet transmission slopes were modelled with 0.2log unit/10 nm, consistent with Hart and Vorobyev (2005) with moderate slopes of *k*=0.15 for SWS2 and *k*=0.12 for Rh2 and LW. The plotted normalized effective photoreceptor sensitivity range is the product of opsin absorbance×droplet transmission, attenuated relative to opsin absorbance (Table S4, Dataset S3 in https://figshare.com/s/dc412ea22f2eca8ff869).

The exponentially decaying visual pigment absorbance spectrum allows calculation of the upper limit of cone sensitivity, sensitivity which is not extended by lateral filtering alone (Bernard, 1979) and can be obtained from the visual Govardovskii template (Govardovskii et al., 2000) at the wavelength intercepting 5% sensitivity.

### Reflectance spectrum of visual marker devices

Detection markers as installed in ski resort stations were sampled and their characteristics are described in Table S1. One marker of each type was measured. The markers were new, as supplied by the manufacturer. A reference blank (spectralon) was used as a baseline before each measurement. Reflectance measurements were carried out using a high-performance spectrophotometer [Cary 5000 Ultraviolet-Visible-Near Infrared (UV-VIS-NIR) spectrophotometer, Agilent] capturing wavelengths every 1 nm from 300 to 780 nm using a photomultiplier tube detector. Most materials were opaque, therefore not transmitting light rays and preventing measurement of light transmission. Two types of reflectance were measured: (i) diffuse reflectance, in which the incident light is scattered in many directions, and (ii) combined diffuse and specular reflectance, which includes both scattered and reflected components. Specular reflection corresponds to the direct, angle-dependent reflection and is generally more perceptible at greater distances than diffuse reflection. We measured each component once separately, yet in most optical situations, both diffuse and specular components contribute simultaneously to the reflected signal.

To quantify the achromatic contrast between different materials of the same object, we computed Michelson contrast values for the mean spectral reflectance of each material surface. For each sampled area, we first extracted the reflectance spectrum (300–780 nm), then computed the mean reflectance value across all wavelengths. This yielded a single scalar per sample, summarizing its overall reflectance intensity, used as input for further contrast analysis. The Michelson contrast (*C*_m_) between two surfaces with reflectance R_1_ and R_2_ was calculated as:
(4)

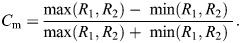
This metric provides a relative measure of contrast that is independent of absolute luminance. We then estimated the inverse contrast value (1/*C*_m_) to compare with the maximum spectral sensitivity of the black grouse (Dataset S4 in https://figshare.com/s/dc412ea22f2eca8ff869). Transparent floats were not included in this analysis as they are composed of a single type of material.

## RESULTS

### Black grouse visual fields

**Fig. 2.**
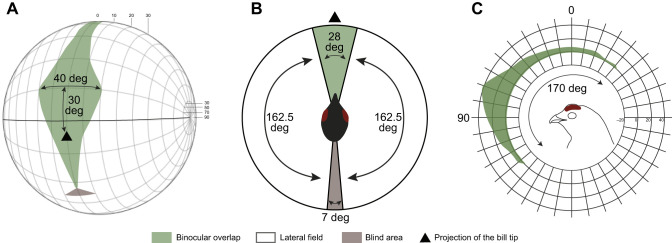
**Visual fields of the black grouse, *Lyrurus tetrix*.** Values represent the mean visual field for two measured individuals. (A) Orthographic projection of the retinal field boundaries from both eyes. A latitude and longitude coordinate system is used with the equator aligned vertically in the median sagittal plane. The bird head is positioned at the centre of a schematic globe, and the projection of the bill tip corresponds to the black triangle. The grid lines are drawn at 10 deg intervals in latitude and at 20 deg intervals in longitude. (B) Section through the horizontal plane corresponding to the bold line in A, and showing the binocular overlap (green), lateral fields (white) and blind spot (brown). (C) Binocular field as a function of vertical elevation in the median sagittal plane. The bird head orientation is shown with the direction of the bill tip. All values are in degrees, and the grid is at 10 deg intervals.

The maximum binocular overlap of the male black grouse covers 40 deg and is positioned 30 deg above the eye bill-tip direction (Fig. 2A). At rest, the black grouse has a field of view covering 353 deg in the horizontal plane, including a frontal binocular overlap of 28 deg, lateral fields of 162.5 deg each, and a blind sector of only 7 deg behind the head (Fig. 2B). The vertical binocular field in the median sagittal plane extends over 170 deg (Fig. 2C), comprising 135 deg above the eye bill-tip direction to 35 deg below it, which also corresponds to a range from 35 deg behind the head to 135 deg in front (Fig. 2C). Above the head (0 deg), the binocular field has a width of 6 deg.

### Axial length and visual acuity

The measured CD from close-up photographs was 8.00 mm for both individuals. The estimated axial length was 13.28 mm, which corresponds to a spatial resolution of 9.81 cpd.

### Contrast sensitivity

**Fig. 3.**
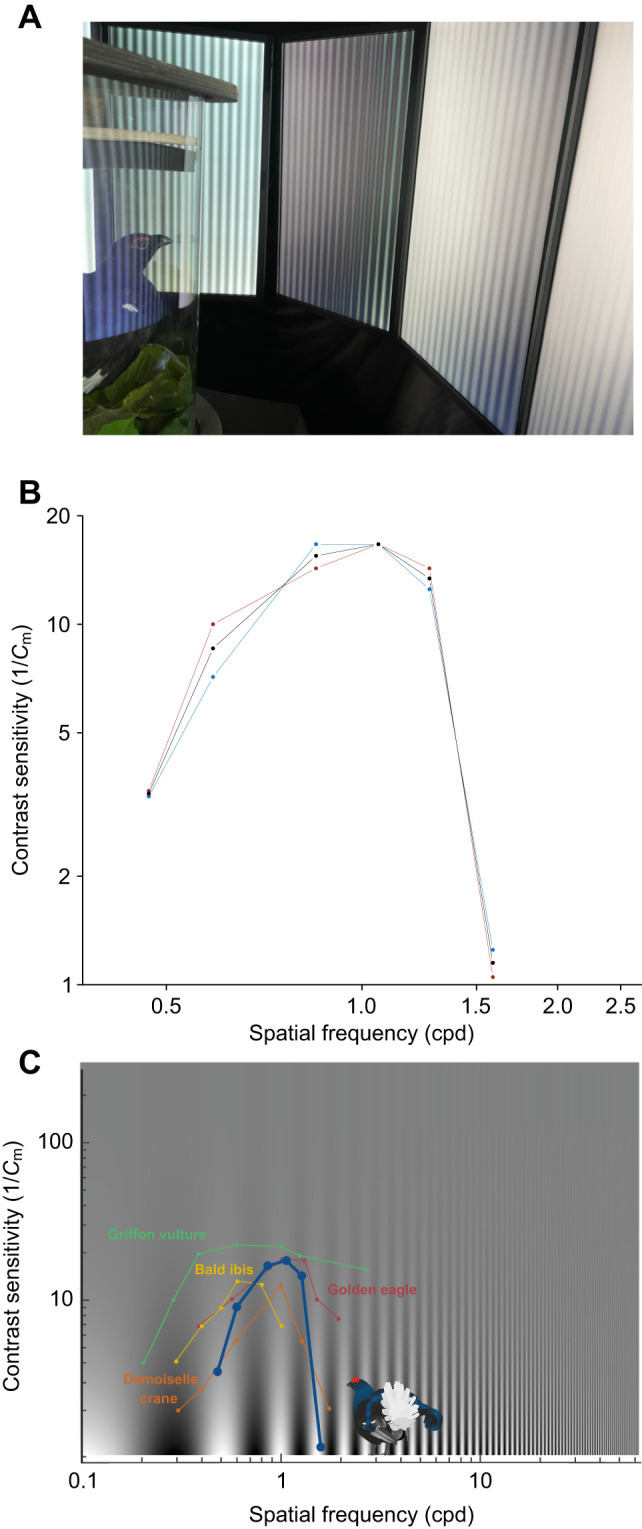
**Contrast sensitivity of the black grouse.** Contrast sensitivity is defined as the inverse of contrast threshold, as a function of spatial frequency. (A) Picture of a black grouse in the apparatus, where the eyes face monitors displaying gradient stripes (note that apparent differences in colour and brightness are due to photographic artifacts; in reality, monitor outputs were identical). (B) Dashed red and blue lines represent contrast sensitivity, expressed as the inverse of the Michelson contrast (1/*C*_m_) values, for individual A and B. The black curve represents the mean. (C) Comparison of the contrast sensitivity between the black grouse (blue curve) and representative species also prone to collisions based on values from Blary et al. (2024). Coloured curves correspond to the following species: red, golden eagle; yellow, griffon vulture; green, bald ibis; orange, demoiselle crane. Logarithmic scales (log_10_) are used in B and C.

To assess the bird's ability to distinguish features in its visual environment, we projected stripe patterns with varying contrast and spatial frequencies. Fig. 3B shows that contrast sensitivity decreased progressively with increasing spatial frequency, corresponding to a smaller distance to object or to an increase in object size. Contrast sensitivity also decreased with decreasing spatial frequency, corresponding to a longer distance to object or a reduction in object size. This typical contrast sensitivity pattern results in an inverse U-shaped curve (Fig. 3B). The estimated optical frequency, i.e. associated spatial resolution of maximum contrast sensitivity, was 0.85 cpd for individual A and 1.06 cpd for individual B. The average optical frequency was 0.95 cpd, corresponding to a maximum contrast sensitivity of 16.67 (Fig. 3B). This sensitivity corresponds to a Michelson contrast (*C*_m_) of 6% (i.e. contrast sensitivity=16.67=1/*C*_m_; *C*_m_=1/16.67≈0.0599 or 5.99%), which means that black grouse can distinguish a signal until it is only 6% brighter than the surrounding background. We then estimated the maximal spatial resolution in a moving environment; that is, the detection limit of the visual system when contrast sensitivity is set to 1 (or *C*_m_=1/1=1=100%), i.e. only a black and white grating with 100% contrast would be just detectable. Based on Fig. 3B, at a contrast sensitivity of 1 (100% contrast), the spatial frequency reaches 1.6 cpd (Fig. 3B).

### Opsin absorbance

**Fig. 4.**
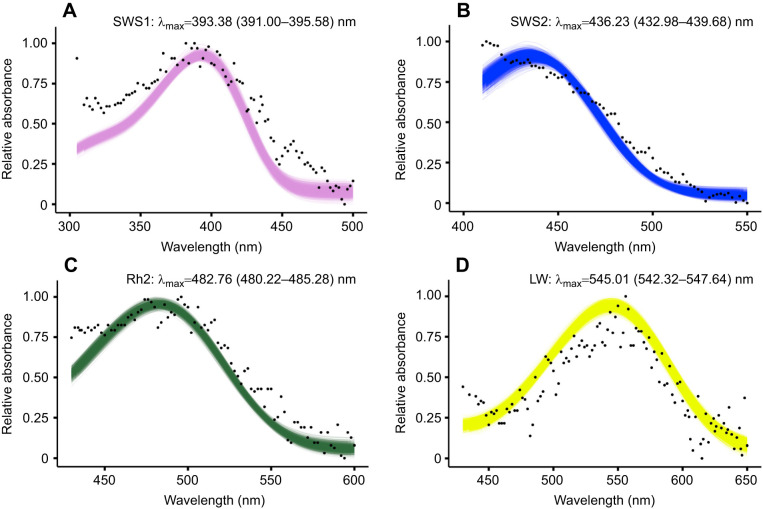
**Absorbance range of the black grouse visual cone opsins purified in complex with 11-cis-retinal.** (A–D) The black dots represent the average absorbance across replicates at every wavelength, whereas the lines represent the curve fitting of individual bootstrap replicates. (A) SWS1 (*n*=7), (B) SWS2 (*n*=6), (C) Rh2 (*n*=3), (D) LW (*n*=7), where *n* is the number of measurements of protein aliquots with active rhodopsin complexes. For each rhodopsin, relative absorbance data were fitted to a visual template with polynomial function analyses computed in R to obtain the estimates of the maximal wavelength sensitivity (λ_max_) following 1000 bootstrap replicates. Values in parentheses represent the lower and upper bounds of the 95% confidence intervals. Accompanying data are provided in Dataset S2 (https://figshare.com/s/dc412ea22f2eca8ff869).

**Fig. 5.**
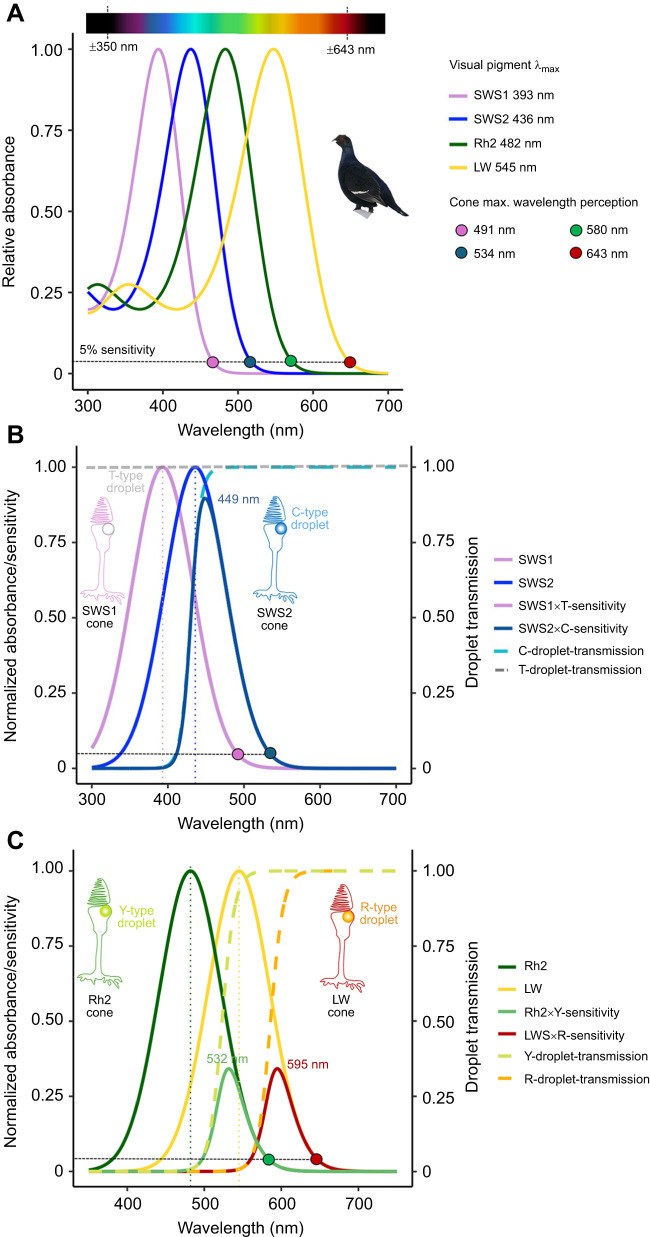
**Visual opsin pigment absorbance range and modelled spectral sensitivity in the black grouse.** (A) The visual pigment absorbance range confers wavelength perception from ca. 350 nm to 643 nm, where opsin absorbance drops to 5% sensitivity (dashed horizontal grey line). (B) Theoretical effective SWS cone spectral sensitivity is calculated as SWS×oil droplet sensitivity. SWS1 cones have transparent T-type oil droplets that transmit light (T=1) and the effective SWS1 cone spectral sensitivity is similar to the opsin-based absorbance range. In SWS2 cones, C-type droplets filter short wavelengths under 430 nm, following the relationship established by Hart and Vorobyev (2005), modelled as a likely shift of the SWS2 peak sensitivity to approximately 449 nm. (C) Theoretical effective Rh2 and LW cone spectral sensitivities. Y- and R-type droplet transmission mid-wavelength of absorbance (λ_mid_) values vary moderately across avian species (Y-type 521–544 nm, R-type: 585–613 nm) and estimates of Y λ_mid_ of 523 nm and R λ_mid_ of 586 nm are used here, based on known microspectrophotometry average values in other Galliformes such as *Gallus gallus* (table 1 in Hart and Vorobyev, 2005), parsimoniously placing the range of effective cone sensitivity from 532 to 580 nm for Rh2 and from 595 to 643 nm for LW. Modelling scripts are presented as the accompanying Dataset S3 (https://figshare.com/s/dc412ea22f2eca8ff869).

We determined the range of absorbance of the four cone opsins (SWS1, SWS2, Rh2, LW) expressed in the black grouse retina as determined via RNA expression profiling. Active rhodopsin complexes were reconstituted *in vitro* by addition of 11-cis-retinal, purified and analysed by UV-VIS spectroscopy to determine their absorbance profiles. These analyses showed that the black grouse SWS1 and SWS2 opsins absorb maximally at a λ_max_ of 393±2 nm (Fig. 4A) and 436±3 nm (Fig. 4B), respectively. We then measured the absorbance of black grouse Rh2 and LW, which displayed maximum sensitivity to light at 482±2.5 nm (Fig. 4C) and 545±2.5 nm (Fig. 4D), respectively. Black grouse cone opsins thus perceive wavelengths from ca. 350 nm to 643 nm (Fig. 5A) when applying a visual template fit with a 5% sensitivity cut-off for the lower and upper absorbance shoulders of SWS1 and LW, respectively.

### Modelled spectral sensitivity

Using the empirical visual pigment λ_max_, which provide the UV-VIS spectral range and maximal wavelength perception (Fig. 5A), we then modelled the effect of oil droplets on theoretical effective photoreceptor sensitivity in SWS, Rh2 and LW cones (Fig. 5B,C). In SWS1 cones, spectral sensitivity corresponds to the visual pigment sensitivity and in SWS2 cones, sensitivity is maximized above 449 nm (Fig. 5B). In Rh2 and LW cones, Y- and R-type oil droplets tend to shift the resulting maximal cone spectral sensitivity to longer wavelengths, which modelling indicates would probably be maximal at 532 nm and 595 nm, respectively (Fig. 5C). Because oil droplet filters do not alter the long-wavelength limb of absorption by the expressed opsin, the maximal wavelength perceived by the LW photoreceptor remains at ∼643 nm, corresponding to the 5% sensitivity cut-off by the descending absorbance shoulder (Fig. 5).

**Fig. 6.**
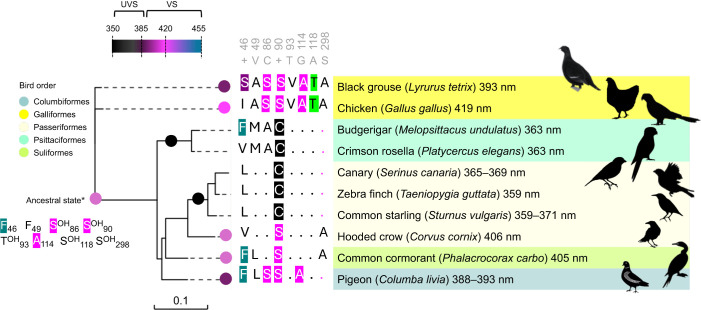
**SWS1 opsin phylogeny of representative bird species with known SWS1 visual pigment maximal absorbance (λ_max_).** Vertebrate SWS1 pigments are divided into two subtypes (UV sensitive, UVS λ_max_ 355–385 nm; violet sensitive, VS λ_max_ 385–455 nm) (Hauser et al., 2014) and different circle colours are used to represent UV-shifted (black) to more violet-shifted light. Maximal absorbance peak values measured from purified visual pigments are indicated after each species name and are taken from Hart (2001) and Hart and Hunt (2007). Key amino acid sites and residue substitutions contributing to avian SWS1 maximal sensitivity (reviewed in Hauser et al., 2014) are indicated along phylogenetic branches, with numbering relative to bovine rhodopsin residue positions (accession number AAA30674). The ancestral avian violet SWS1 sensitivity was inferred from Hagen et al. (2023). OH refers to amino acids bearing a hydroxyl group. Dots in the alignment correspond to a conserved residue with the consensus sequence in grey (+ indicates a variable site). NCBI sequence accession numbers: chicken NP_990769, budgerigar NP_001298010, canary NP_001289029, zebra finch NP_001070172, common starling XP_014746280, hooded crow XP_039427152, common cormorant ABS86975.1, pigeon NP_001269750.

Avian SWS1 opsins are divided into ultraviolet (λ_max_<385 nm) and violet-sensitive (λ_max_>385 nm) types (Hauser et al., 2014), and the black grouse visual pigment, based on its λ_max_ is classified in the violet range but nevertheless absorbs a small fraction of UV light (Figs 3 and 4A). We aligned and compared SWS1 opsin sequences between the black grouse and additional representative avian species with known SWS1 opsin absorbance, highlighting amino acid residues at positions previously found to be involved in spectral tuning (Fig. 6) (Hauser et al., 2014; Hagen et al., 2023). The black grouse SWS1 opsin lacks the cysteine residue present in all true UV-sensitive opsins with λ_max_<370 nm (Fig. 6) and shares different degrees of sequence similarity at these spectral positions with the chicken (λ_max_ 419 nm) and pigeon (λ_max_ 393 nm) SWS1 opsins.

### Reflectance spectra of visual markers

**Fig. 7.**
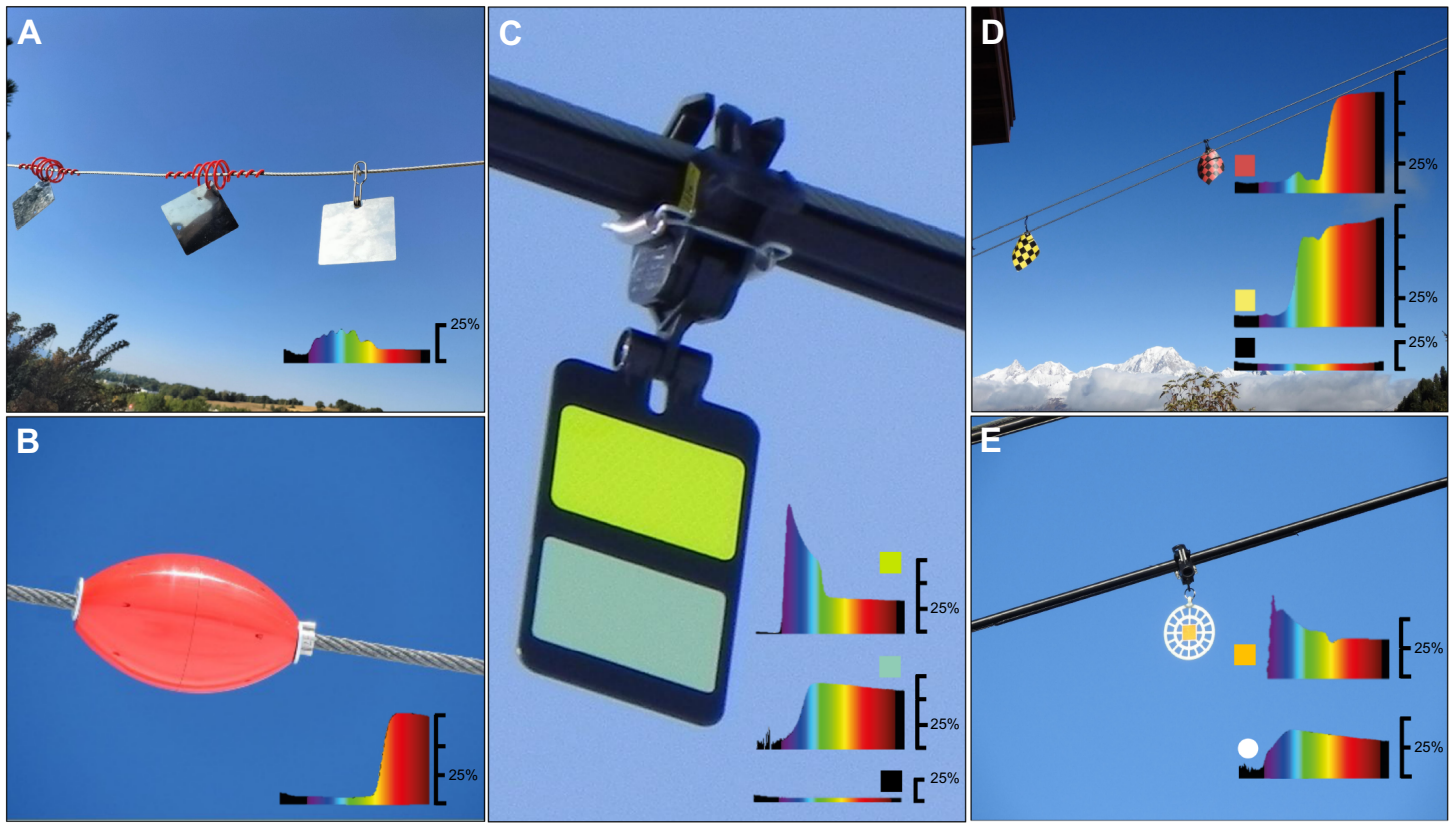
**Examples of aerial cables equipped with visual markers and their diffuse reflectance profiles.** See also Table S1 for more designs, and Figs S1–S8 for a comparison between diffuse and specular reflectance. Photographs show (A) a stainless-steel plate (8×8 cm) for aerial cables used for avalanche control; (B) an opaque float (7.5×4.5 cm) installed on ski lift safety ropes; (C) a black Firefly (15×9 cm) installed on chairlifts; (D) a patterned flag (16×16 cm) for aerial cables used for avalanche control; and (E) a white orange Birdmark (diameter 13.5 cm) installed on chairlift and ski lift safety ropes. Insets: reflectance spectra from 300 nm to 780 nm (*x*-axis). Reflectance within the human visible range is colour coded from violet (400 nm) to red (750 nm). Reflectance outside this range (<400 nm and >750 nm) is shown in black. Horizontal marks on scale bars correspond to 25% reflectance each.

Visual markers show spectral reflectance within the visible spectrum, and a few materials show reflectance in the ultraviolet range below 400 nm and long-wavelength light above 750 nm (Fig. 7; Figs S1–S8). For most materials except the polished stainless-steel plate (Fig. S7), the specular and diffuse reflection components are nearly identical (Figs S1–S8). Because of the variety of existing designs, some materials show relative reflectance values exceeding 100% owing to fluorescent elements.

### Internal achromatic contrasts of visual markers

**Fig. 8.**
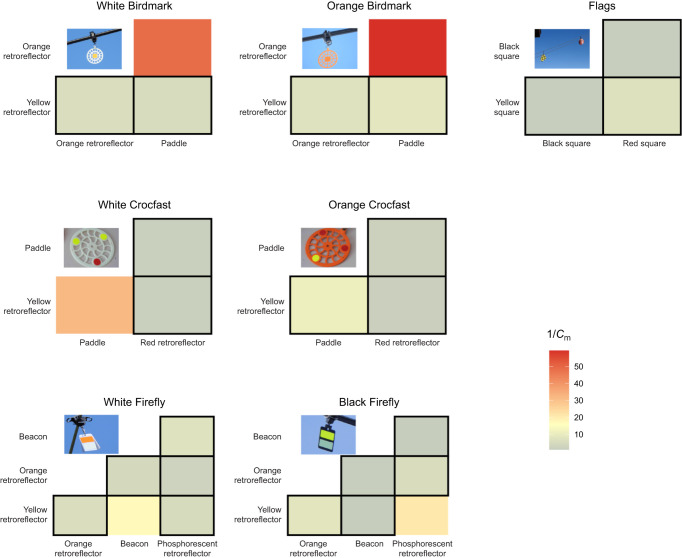
**Heatmap representation of internal contrasts in visual markers composed of multiple reflective surfaces.** Contrasts are calculated as the inverse of the Michelson contrast threshold. Squares outlined in black indicate cases where contrasts fall below the maximum contrast sensitivity of the black grouse (1/*C*_m_≤16.67), suggesting that the visual system can perceive the difference in brightness between components of the visual marker, which exceeds the smallest brightness difference detectable by the black grouse visual system at its peak critical flicker fusion (CFF) frequency; that is, when the visual system is most sensitive to changes in brightness. See also Dataset S4 (https://figshare.com/s/dc412ea22f2eca8ff869).

Internal achromatic contrasts between adjacent reflective materials used in white Birdmark, orange Birdmark, black Firefly, white Crocfast, orange Crocfast, flags and white Firefly visual markers (Table S1; see Dataset 4 in https://figshare.com/s/dc412ea22f2eca8ff869) are presented in Fig. 8. Most internal material combinations exhibit values below the black grouse achromatic contrast detection limit (1/*C*_m_≤16.77), indicating high internal achromatic contrast. Low-contrast combinations occurred for the orange retroreflector–paddle interface on Birdmarks, the yellow retroreflector–paddle interface on the white Crocfast, and the white beacon–yellow retroreflector interface on the white Firefly (see Dataset 4 in https://figshare.com/s/dc412ea22f2eca8ff869). In the black Firefly, low internal contrast was observed between the yellow and phosphorescent retroreflectors, while other internal contrasts were within detection limits.

## DISCUSSION

Constraints in bird visual perception have been suggested as one of the factors contributing to variable risks of collisions with human-made structures (Martin, 2011a, 2022; Martin and Banks, 2023). In this study, we examined visual characteristics of the black grouse, specifically its visual field, contrast sensitivity, corneal size (and the estimated visual acuity), as well as visual pigment spectral range to gain a better understanding of its multifaceted vision, and discuss how these factors may affect the perception of obstacles and visual markers.

### Visual fields

Avian visual fields reflect ecological differences, such as in navigation and foraging, parental care or predator detection, and vary significantly among avian species (Martin, 2017a). The visual field configuration of the black grouse shows that this species has an exceptionally extended visual field in both the horizontal and vertical planes. From more than 250 avian species studied to date (Martin, 2017a; Potier et al., 2018a, 2023; Cantlay et al., 2023), only four have a blind visual sector behind the head smaller than that of the black grouse (7 deg), i.e. the Eurasian woodcock (*Scolopax rusticola*) (Martin, 1994) and three duck species, the mallard (*Anas platyrhynchos*), the Northern pintail (*Anas acuta*) and the pink-eared duck (*Malacorhynchus membranaceus*) (Martin, 1986; Guillemain et al., 2002; Martin et al., 2007).

In the context of avian collision, both the binocular overlap and vertical extent of the binocular field are important parameters. The binocular overlap is thought to function primarily in the detection of symmetrically expanding optic flow-fields that provide almost instantaneous information on the direction of travel and time to contact a target, whereas the vertical extent defines whether birds can see in the direction of their flight path (Martin, 2009, 2017a, 2022; Martin and Portugal, 2011; Martin et al., 2012).

The black grouse frontal binocular field covers 28 deg in the horizontal plane, in agreement with typical avian values (20–40 deg) (Martin, 2017a), and a wide maximal binocular overlap (Fig. 2A,C), which is similar to that of other visually guided ground forager species such as the Southern caracara (*Caracara plancus*) (Potier et al., 2017). Together, the binocular field of the black grouse is well adapted to its ground foraging behaviour.

The vertical extent of the binocular field allows black grouse to gain a fairly comprehensive visual coverage of the celestial hemisphere, and the ability to see the way ahead irrespective of their head position. This configuration should therefore enable black grouse to detect aerial objects. However, adult males develop enlarged combs during the breeding season (Rintamäki et al., 2000), which may partially reduce the vertical extent of the binocular field. This potential reduction in older males, particularly during the breeding season, warrants further investigation across developmental stages, including comparisons with females. In contrast to the black grouse, many large raptors, such as some eagles, vultures or hawks (Accipitriformes) may fail to see the way ahead (Martin et al., 2012; Portugal et al., 2017; Potier et al., 2018a). Hence, while in flight, visualizing the ground below for food, their limited field of view would provide comparatively no or only intermittent information about the flight path ahead (Martin et al., 2012).

Thus, the finding that black grouse can detect objects in a wide horizontal plane and also in the vertical plane is informative to directly optimize aerial human-made structures. Optimizations include modifications of, for example, an object's size or spacing, its internal contrasts or contrast relative to the environmental background, or its spectral properties (colours), and/or using visual markers (Martin, 2022) to improve the detection of an object.

### Spatial resolution, visual acuity and detection distance

Avian spatial resolution is highly variable, from as low as 4 cpd in the barn owl (*Tyto alba*) to as high as 142 cpd in the wedge-tailed eagle (*Aquila audax*) (Harmening et al., 2009; Reymond, 1985). The black grouse has a spatial resolving power of 9.81 cpd, which is relatively low. Its visual acuity, estimated from its eye size, is nevertheless very similar to that of closely related species in the Order Galliformes, ranging from 9.7 cpd in the Japanese quail (*Coturnix japonica*) to 13 cpd in the sharp-tailed grouse (*Tympanuchus phasianellus*) (Lisney et al., 2012). Although additional precision may be gained through approaches based on photoreceptor density or behavioural assays, these results show that eye size remains a reliable proxy for spatial resolution (Kiltie, 2000; Caves et al., 2024) and can provide estimates comparable to those obtained with operant conditioning (Potier et al., 2016).

Spatial resolution informs at which distance black grouse can detect objects in optimal environmental conditions, that is (i) with no air distortion and a clear sky, (ii) for a black object on a white background (i.e. high contrast scenario), (iii) at high daylight levels and (iv) with the bird visually fixing the object with its acute centre of vision (Land and Nilsson, 2012; Martin, 2022). Under optimal conditions, the distance at which an object can be detected and avoided depends on three factors: the spatial resolution, reaction time (*t*_react_) and flight speed (*v*) (Martin, 2022). In birds, it is reasonable to consider that a minimum *t*_react_=2 s is necessary to initiate a change in flight path (Martin, 2022). Using the allometric relationship between body mass and flight speed (*v*) presented by Alerstam et al. (2007), the mean body mass of black grouse (1068.7 g; Dunning, 2007) predicts a typical *v* of 16.04 m s^−1^. If the object only needs to be visible during reaction, the minimum detection distance (*D*) of an object while in flight is *D*=2*v*. Therefore, the minimal distance at which a flying black grouse could resolve two adjacent objects sufficiently early to adjust its flight path in order to avoid collision is estimated as 2×16.04=32 m. This represents a conservative lower value that may increase with bird motion, atmospheric turbulence or directional lightning conditions typical of mountain environments.

From this minimal detection distance of 32 m, the spacing (*d*) between two objects (e.g. two adjacent visual markers) while actively flying can be estimated using the trigonometric relationship between angle and detection distance as follows: *d*=2*D*tan(θ/2), where θ is the binocular overlap angle in radians. This indicates that when black grouse fly at the same altitude as an object (e.g. a visual marker), the minimal spacing between two adjacent markers for them to be visible to the black grouse is *d*=2×32tan(28 deg)≈16 m.

Under optimal conditions and considering the visual acuity as a proxy of corneal diameter, the minimum width of an object (*w*) to meet the spatial-frequency limit of one cycle can be estimated using:
(5)

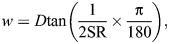
where SR is spatial resolution. A factor of 2 is applied because visual acuity is expressed in cpd, and one cycle corresponds to two resolvable bars; birds can therefore discriminate two objects within a single cycle. With a spatial resolution of 9.81 cpd, the minimal width (*w*) of an object to be detected at 32 m is:
(6)

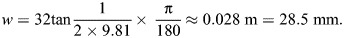
A minimum width of 28.5 mm is 4–5 times larger than the smallest object detectable by an average human eye with its spatial resolution of 40–50 cpd (Martin, 2017a). In addition, in a natural scene, optimal conditions are rarely reached; spatial resolution declines up to 5-fold with decreasing light levels, e.g. on a cloudy day, or at dusk and dawn (Land and Nilsson, 2012; Martin, 2017a, 2022). To compensate for this lower resolution, the object width should linearly be increased 5 times, therefore reaching a minimum width of 142.5 mm, a width currently only met by flag designs in the markers tested (15 cm×15 cm, Fig. 7D; Table S3).

### Contrast sensitivity

The maximum contrast sensitivity of the black grouse (16.67) is in the middle range compared with that of various birds, e.g. from 4.8 in the Bourke's parrot (*Neopsephotus bourkii*) to 31 in the American kestrel (*Falco sparverius*) (Hirsch, 1982; Lind et al., 2012; Blary et al., 2024). Values in birds are typically lower than contrast sensitivity observed in other vertebrates such as most rodents (20) or humans (close to 200) (De Valois et al., 1974; Birch and Jacobs, 1979). As the black grouse maximum contrast sensitivity is ∼10 times lower compared with that of the human eye, a dark aerial cable or a dark lift structure against a dark green forest background, or a visual marker against a snowy background (Fig. 1) would be more difficult for a black grouse eye to detect than for a human eye. Consequently, choosing markers with high internal object contrasts (Fig. 8) as well as high contrast with the surrounding environment (Martin, 2022) should further aid black grouse in detecting aerial infrastructures under variable light conditions.

### Spectral range

The spectral characteristics of the black grouse visual opsin pigments align with avian pigment sensitivities (reviewed in Hagen et al., 2023), and combined its four avian cone opsins absorb wavelengths of light spanning from near-ultraviolet to red from ca. 350 to 643 nm. Whereas transmission properties of cone oil droplets in the black grouse are not yet known, models based on other galliform species are informative to infer the black grouse effective spectral range (Fig. 5). In SWS1 cones, transparent T-type oil droplets do not absorb light (Hart and Vorobyev, 2005), so maximal spectral sensitivity aligns primarily with the SWS1 visual pigment λ_max_. Differences in absorbance characteristics of the avian ocular media can also tune UV sensitivity as the cornea typically also filters a portion of UV light, albeit with large interspecific variation among species, i.e. low and high cut-offs vary between ∼315 and 370 nm (reviewed in Hart and Vorobyev, 2005). The violet-sensitive black grouse SWS1 pigment, with an absorbance peak around 393 nm, would probably be capable of detecting some signals in the UV range, even in the presence of substantial corneal filtering of short-wavelength light. Behavioural research has shown that black grouse preferentially select naturally UV-reflecting berries (Siitari et al., 2002); however, because these fruits also differ in reflectance across the visible spectrum, this preference cannot be attributed exclusively to UV cues. Nevertheless, the results are consistent with the ability of black grouse SWS1 cones to support perception of at least long-wavelength UV light, although direct characterization of lens and corneal UV transmission in this species is still needed. Differences in opsin λ_max_ are largely contributed by molecular variation at tuning spectral sites and have been shown to influence colour perception in vertebrates (Hauser et al., 2014; Hagen et al., 2023). The ecological consequences of small variations within avian violet-sensitive pigments, however, remain to be studied in more detail.

Together with the black grouse SWS2 pigment absorbance, the modelled spectral sensitivity of SWS2 cones (λ_max_ 449 nm) after C-filtering aligns closely with *in vivo* measurements in related galliform bird species, such as the Japanese quail (*Coturnix japonica*, λ_max_ ∼450 nm; Bowmaker et al., 1993) or domestic chicken (*Gallus gallus*, λ_max_ ∼450 nm; Bowmaker et al., 1997), indicating that black grouse SWS2 cones are primarily sensitive to wavelengths above 400 nm up to 534 nm. Rh2 cones are sensitive from above 500 nm to 580 nm with maximal spectral sensitivity predicted at 532 nm after Y-filtering. 580 nm effectively defines the upper theoretical limit for opponency between Rh2 and LW cones and therefore parsimoniously sets the functional limit for chromatic colour discrimination, which based on our values of pigment absorbance and sensitivity modelling in black grouse is restricted from the blue to yellow spectral range. The LW cones have a predicted spectral peak sensitivity at 595 nm after R-filtering, and an upper sensitivity limit at 643 nm, defined by the LW pigment, allowing black grouse to perceive the brightness of orange and red objects. However, because the chromatic contrast is limited by Rh2 sensitivity, most wavelengths above ∼600 nm are probably not distinguished as colours, although achromatic (brightness) perception remains effective up to the LW 5% sensitivity cut-off at ∼643 nm. Compared with humans, whose MW and LW pigment sensitivities peak at 535 nm and 564 nm (Kelber, 2019), respectively, black grouse are thus less capable of chromatically discriminating red–orange hues.

### Internal contrasts of visual markers

For most visual markers, internal achromatic contrast between different materials was generally below the black grouse maximal sensitivity contrast of 16.67 (Fig. 8); in other words, most of these visual features have enough brightness contrast to be perceived by the black grouse visual system. Conversely, specific combinations of adjacent materials may not meet the necessary contrast threshold for detection by black grouse. The orange retroreflector on Birdmarks, but not the yellow one, exhibited a low achromatic contrast (i.e. a high 1/*C*_m_) with the paddle, a contrast which falls outside of the limits of sensitivity for the black grouse. Similarly, the achromatic contrast observed for the yellow retroreflector and the paddle on the white Crocfast appears outside the contrast detection limits. For the white Firefly, the white beacon lacked sufficient contrast with the adjacent yellow retroreflector. For the black Firefly, both the yellow and phosphorescent retroreflectors showed low achromatic contrast; however, this was partially mitigated by the presence of the black beacon, which provides a degree of visual separation between the elements.

### Detection abilities of visual markers and considerations for future optimization

Across a large diversity of materials used to build visual markers currently in use in ski areas in the French Alps, we found that all tend to reflect visible wavelengths of light in the range of perception of the black grouse. Wavelengths shorter than 400 nm are rarely reflected overall, and some of the red visual markers include long-wavelength reflectance above 650 nm, presumably beyond the limits of black grouse perception.

For the black grouse, a marker may thus not benefit from chromatic contrasts above 600 nm (i.e. adjacent yellow and orange, or orange and red patterns would be inefficient). Conversely, visual markers would provide strong chromatic contrasts if they reflect light from 400 nm to 580 nm (i.e. from violet/blue to yellow/orange spectra), and potentially also achromatic signals from long-UV reflectance (∼370–400 nm), although the latter would need to be tested experimentally. In the sandhill cranes (*Grus canadensis*), for which the SWS1 pigment of a closely related *Grus americana* species has a λ_max_ at 404 nm (Porter et al., 2014), night collisions with power lines decreased by 98% after the introduction of UV lamps emitting short wavelengths (380–390 nm) on power poles (Dwyer et al., 2019), suggesting some near-UV sensitivity, similar to the black grouse. Although black grouse are mainly diurnal and their flight behaviour differs from that of cranes, increased UV contrast could be considered in future modelling or field studies to assess their perception against natural backgrounds (Baasch et al., 2022) and under varying natural environmental light.

Chromatic contrast and spectral sensitivity are thus important to consider in collision contexts (Fernández-Juricic et al., 2011; Zheng et al., 2022), and greater differences in the reflectance spectra of spatially adjacent materials correspond to higher internal contrasts, which several visual markers readily possess, such as the Crocfast, flag and Firefly designs (Fig. 8). In line with our observations, high-contrast striped coloured patterns have recently been shown to reduce bird approaches on wind turbines; interestingly, this is possibly due to a resemblance to unattractive aposematic patterns (Hancock et al., 2025 preprint).

In addition to chromatic contrasts, luminance is a highly reliable parameter across different light levels (Land and Nilsson, 2012) and at longer distances because the achromatic spatial resolution is generally higher than the chromatic one (Lind and Kelber, 2011; Potier et al., 2018b). A high internal contrast would both increase visual discrimination against different types of background and improve detection under variable light conditions. Finally, as birds rely on their binocular field to assess the direction of travel and time to contact with an object in their flight path, two adjacent visual makers should be just visible in the bird's binocular field (Martin, 2022). In the case of the black grouse, adjacent markers should be placed no further apart than 16 m and have a minimal width of 142 mm to ensure continuous perception within the binocular field while in flight. This study provides valuable sensory insights applied to the issue of black grouse collisions with aerial anthropogenic infrastructures. To robustly link these visual sensory mechanisms to collision-related mortality will ultimately require field-based ecological validation of cable detection behaviour with and without visual markers.

## Supplementary Material

10.1242/jexbio.250727_sup1 Supplementary information
