# Supplementary material for: Exploring the visual system of the black grouse (Lyrurus tetrix): combining experimental and molecular approaches to inform strategies for reducing collisions
Source: J Exp Biol. 2026 Apr 24;229(8):jeb250727. doi: 10.1242/jeb.250727 (PMC13143217; doi:10.1242/jeb.250727)
Supplement: Supplementary information [file jexbio-229-250727-s1.pdf]

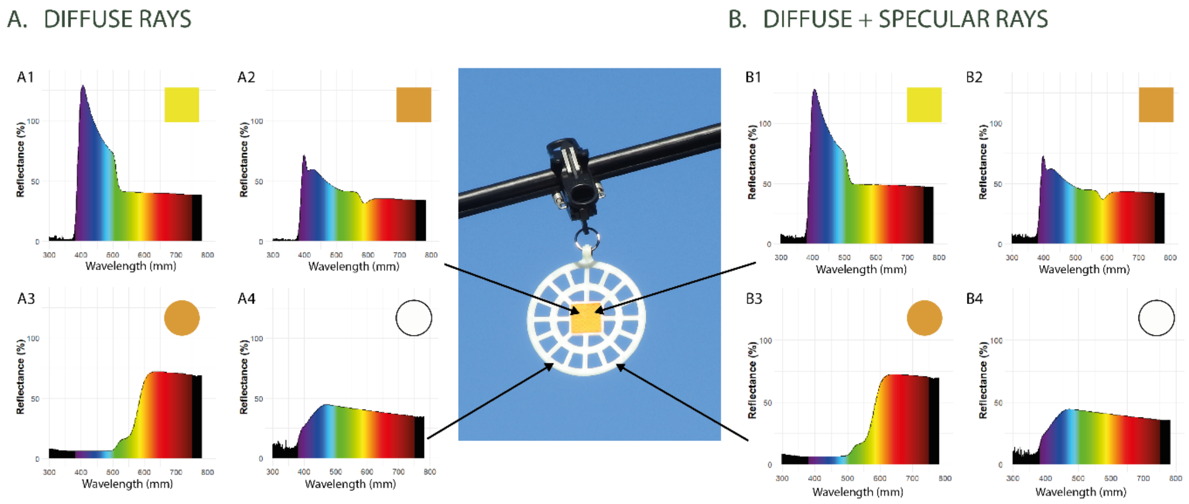

Fig. S1. Diffuse and specular reflection of Birdmarks. A1 and B1 correspond to the yellow retroreflector, A2 and B2 correspond to the orange retroreflector, A3 and B3 correspond to an orange paddle, and A4 and B4 correspond to a white paddle.

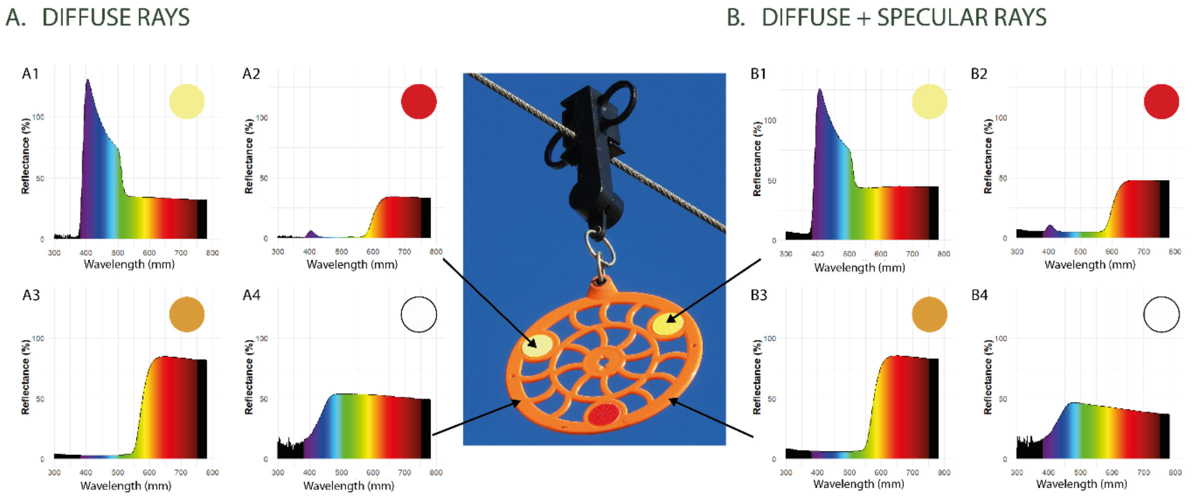

Fig. S2. Diffuse and specular reflection of Crocfast. A1 and B1 correspond to the yellow retroreflector, A2 and B2 correspond to the red retroreflector, A3 and B3 correspond to an orange paddle, and A4 and B4 correspond to a white paddle.

Journal of Experimental Biology • Supplementary information

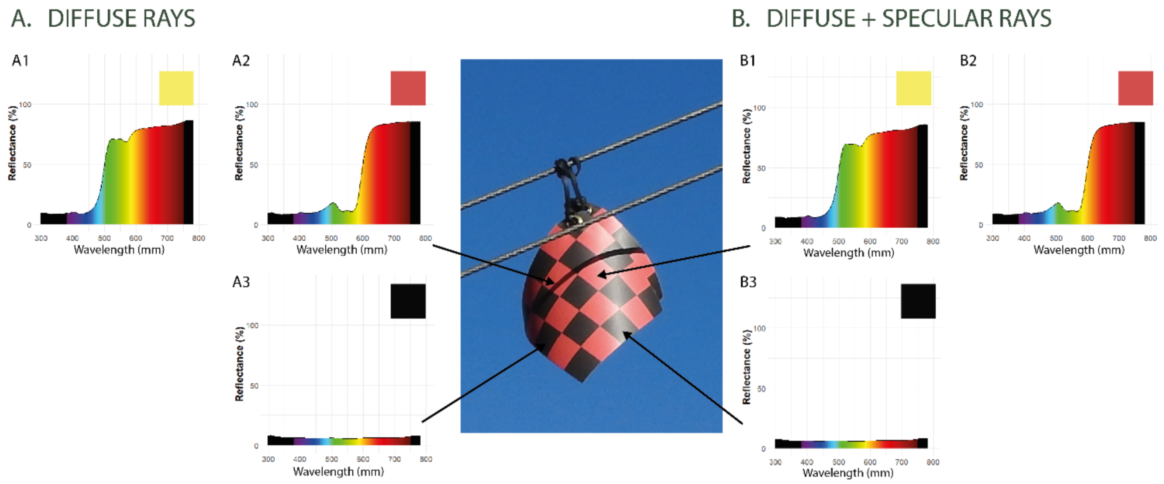

Fig. S3. Diffuse and specular reflection of Flags. A1 and B1 correspond to a yellow square, A2 and B2 correspond to a red square, and A3 and B3 correspond to a black square.

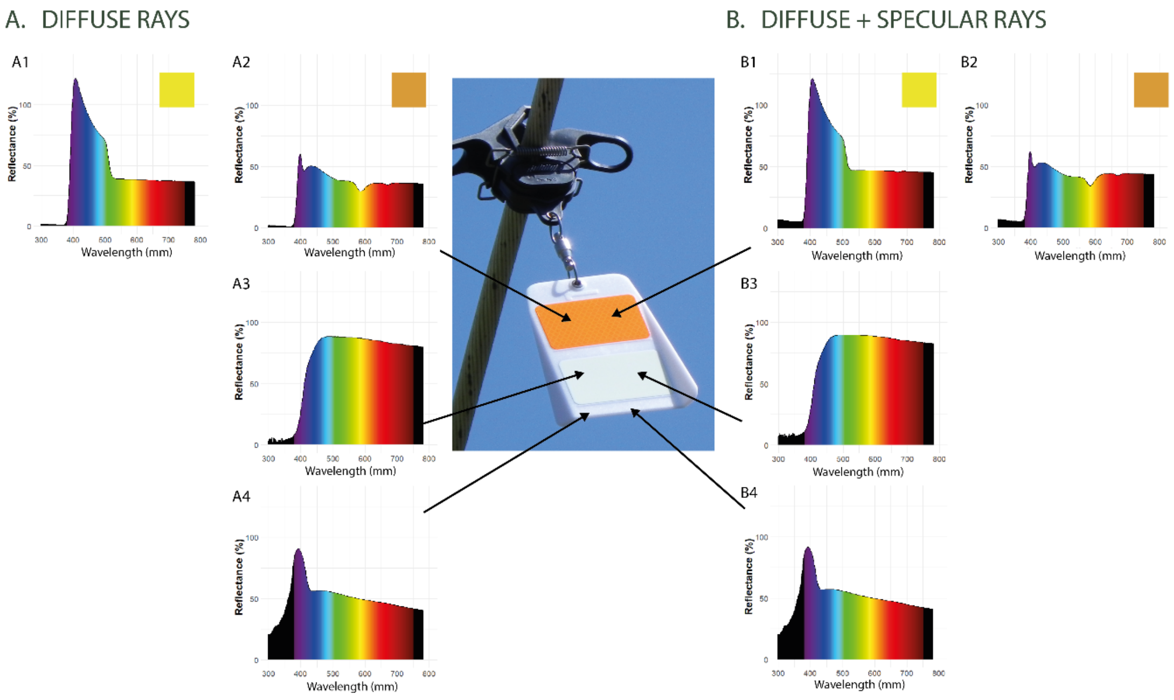

Fig. S4. Diffuse and specular reflection of white Fireflies. A1 and B1 correspond to the yellow retroreflector, A2 and B2 correspond to the orange retroreflector, A3 and B3 correspond to the phosphorescent retroreflector, and A4 and B4 correspond to a white beacon.

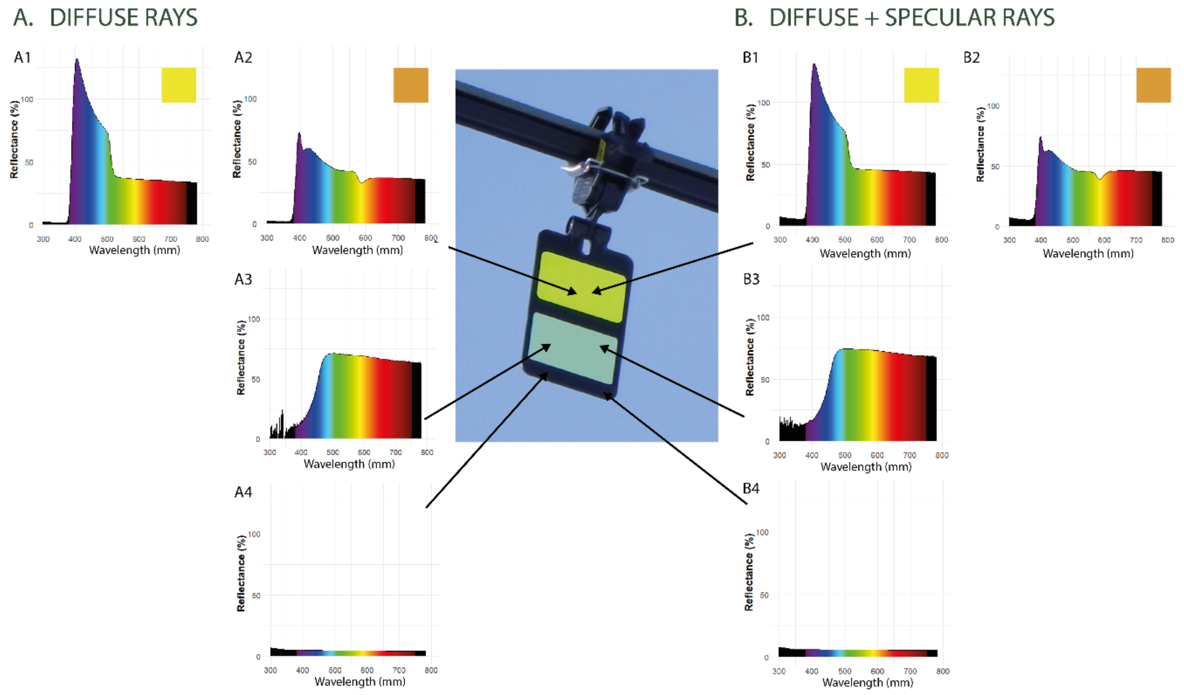

Fig. S5. Diffuse and specular reflection of black Fireflies. A1 and B1 correspond to the yellow retroreflector, A2 and B2 correspond to an orange retroreflector (not shown). A3 and B3 correspond to the phosphorescent retroreflector, and A4 and B4 correspond to a black beacon.

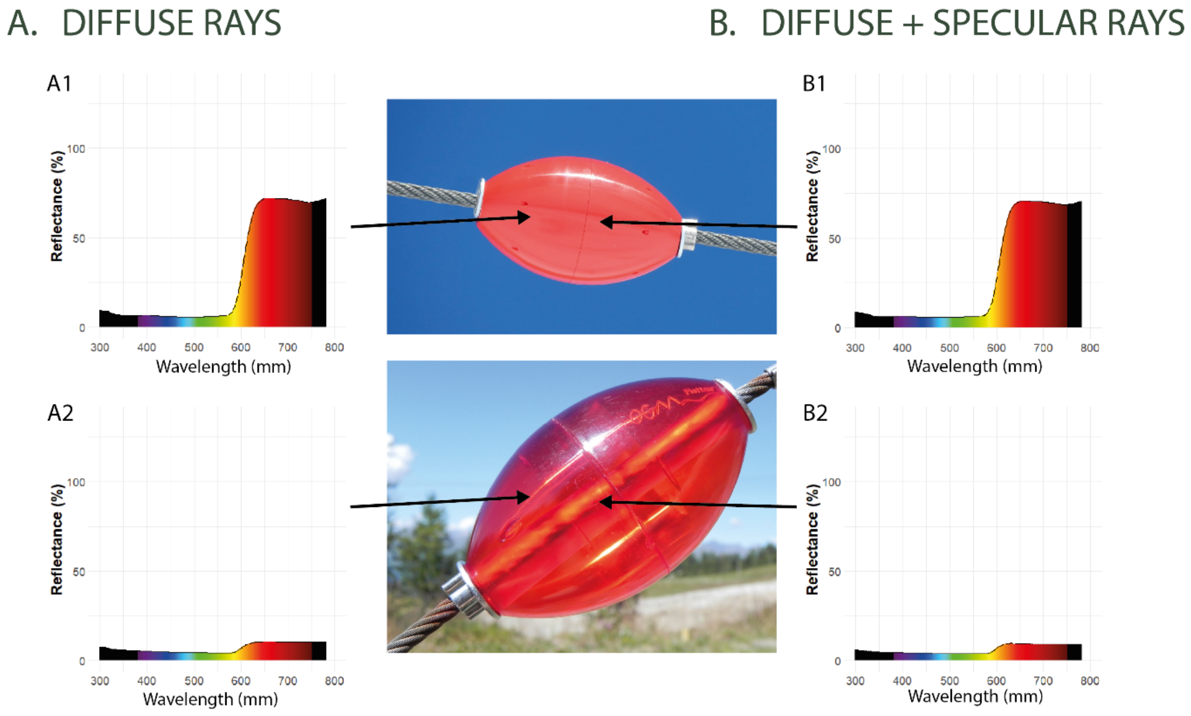

Fig. S6. Diffuse and specular reflection of Floats. A1 and B1 correspond to a transparent float, and A2 and B2 correspond to an opaque float.

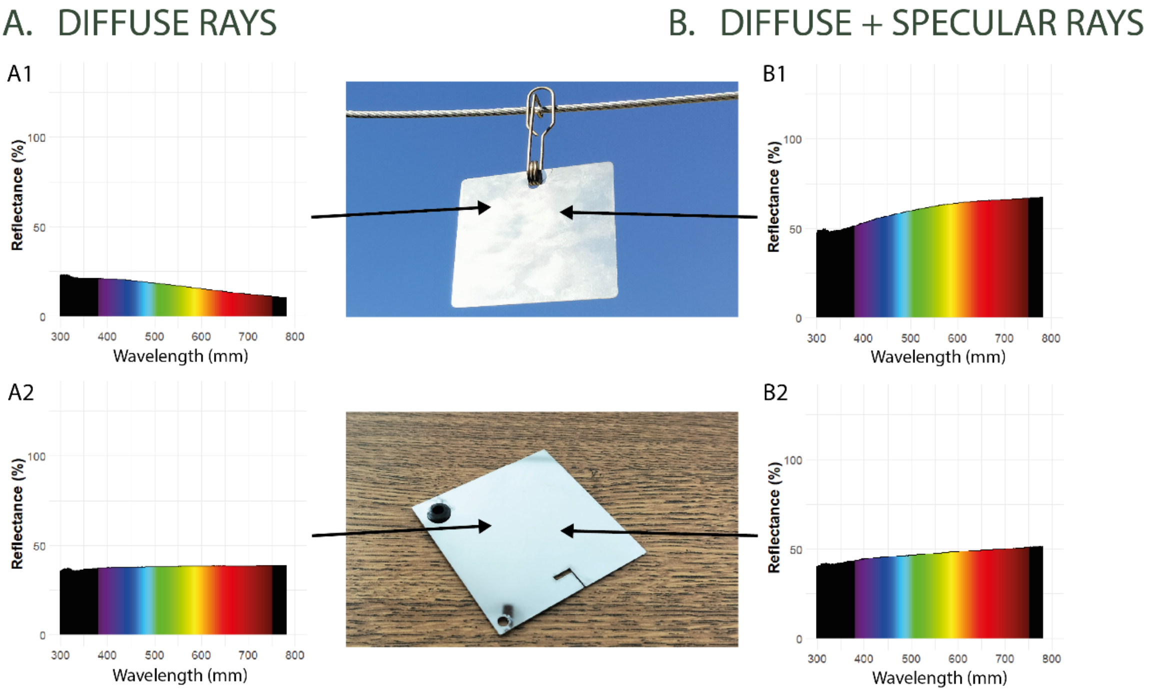

Fig. S7. Diffuse and specular reflection of stainless-steel plates. A1 and B1 correspond to a shiny plate, and A2 and B2 correspond to a matte plate.

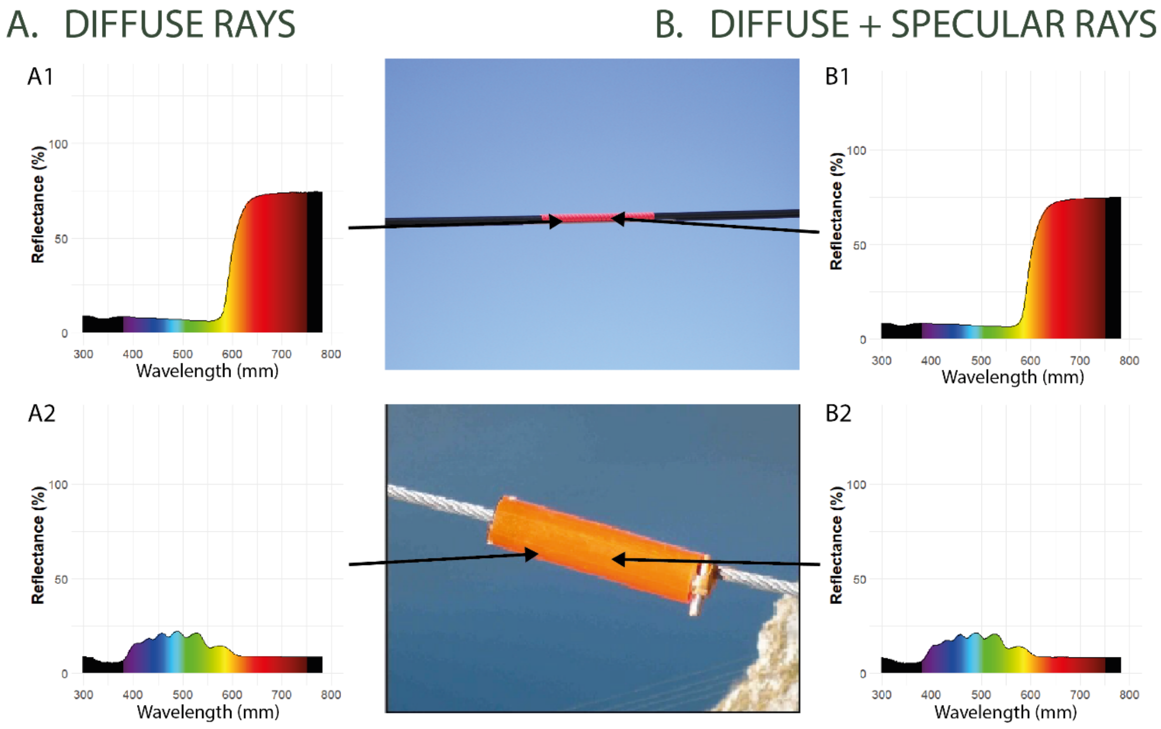

Fig. S8. Diffuse and specular reflection of spirals and fluorescent tubes. A1 and B1 correspond to a red spiral, and A2 and B2 correspond to a fluorescent tube.

Table S1. Visual marker devices.

Types retired or experimental	Photograph
Spirale avifaune<br>Installation on the multipair of chairlifts<br>Size: 30 cm x 4 cm<br>*(product retired)*	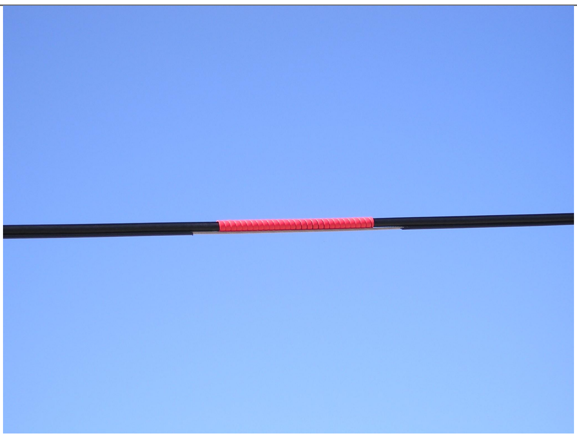
Transparent float<br>Installation on safety rope of ski lifts<br>Size: 7.5 cm X 4.5<br>*(product retired)*	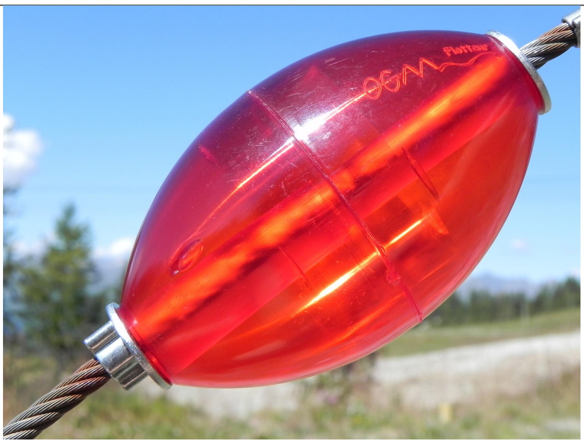
Crocfast (test)<br>Size: Racket diameter = 13.5 cm<br>*(product retired)*	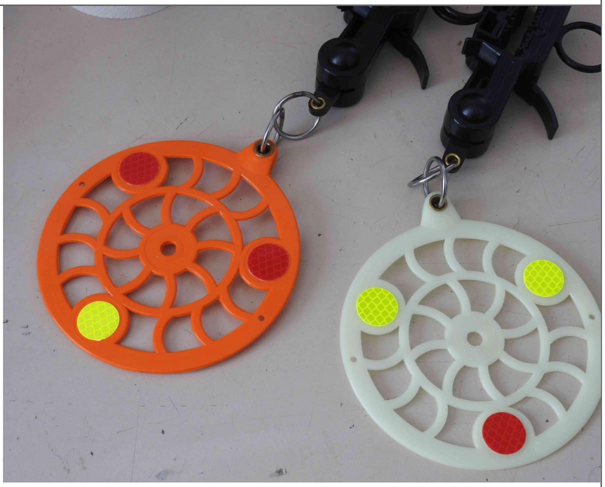

Journal of Experimental Biology • Supplementary information

Fluorescent tube<br>To equip guy cables<br>Size: 15 cm x 3.5 cm<br>*(Experimental, not in use yet*)	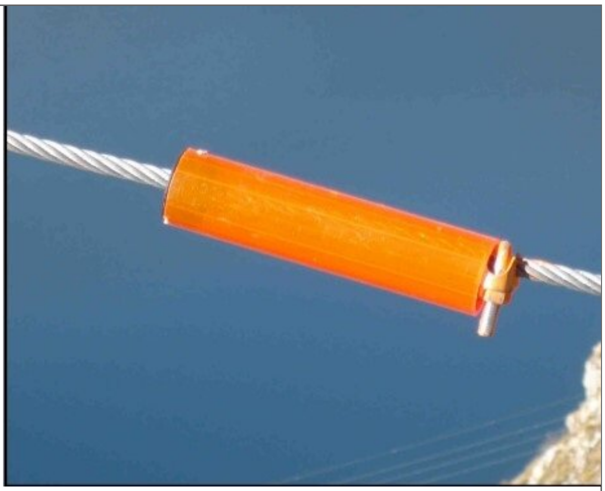
Shiny stainless-steel plate<br>To equip aerial cables for transporting explosives<br>Size: 8 x 8 cm<br>*(Experimental, not in use yet*)	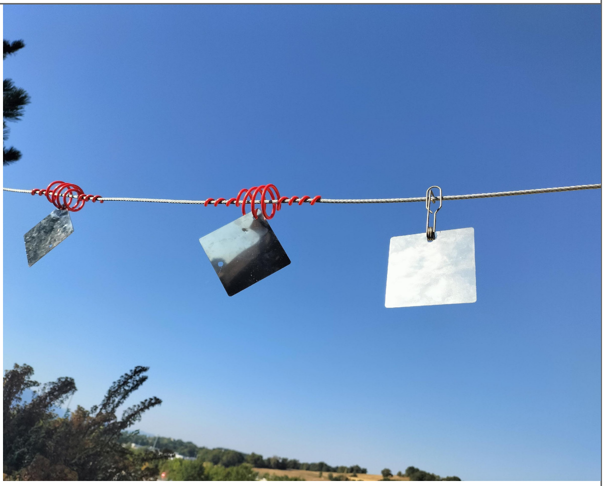
Matte stainless-steel plate<br>Size: 8 x 8 cm<br>*(Experimental, not in use yet*)	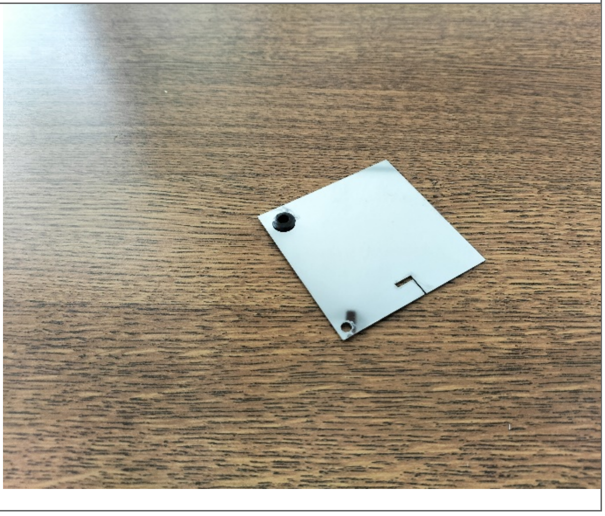

Journal of Experimental Biology • Supplementary information

Flag<br>To equip aerial cables for transporting explosives<br>Size: 16x16 cm<br>*(Experimental, not in use yet*)	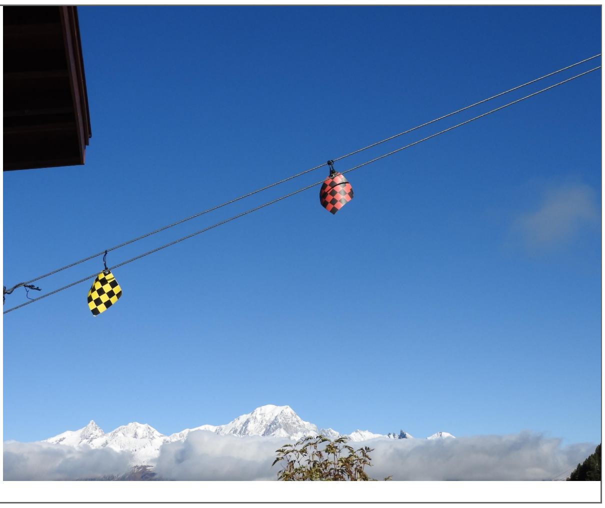

**Types in use**	**Photograph**
Opaque float (OGM Flotteur)<br>Installation on safety rope of ski lifts<br>Size: 7.5 cm X 4.5<br>*(manufacturer: Tool Investissements)*	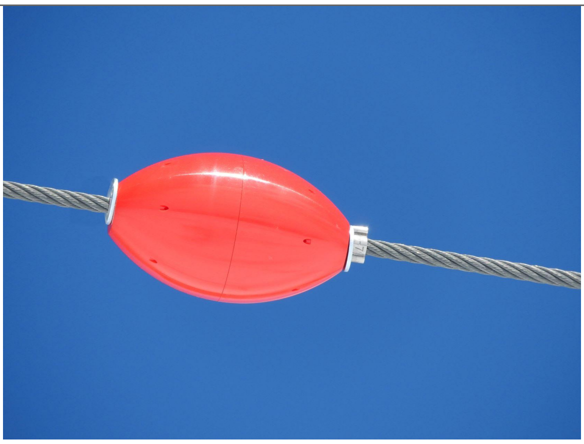
White Birdmark<br>(stores light that is reflected at night)<br>Installation on the multipair of chairlifts, gondola and on safety rope of ski lifts<br>Installation recommendation:<br>Alternation White Birdmark and Orange Birdmark<br>Size: Racket diameter = 13.5 cm<br>*(manufacturer: Hammarprodukter)*	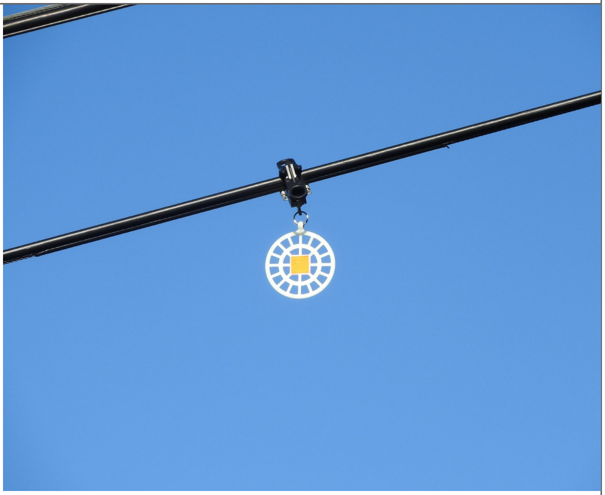

Orange Birdmark<br>Installation on the multipair of chairlifts, gondola and on safety rope of ski lifts<br>Installation recommendation:<br>Alternation White Birdmark and Orange Birdmark<br>Size: Racket diameter = 13.5 cm<br>*(manufacturer: Hammarprodukter)*	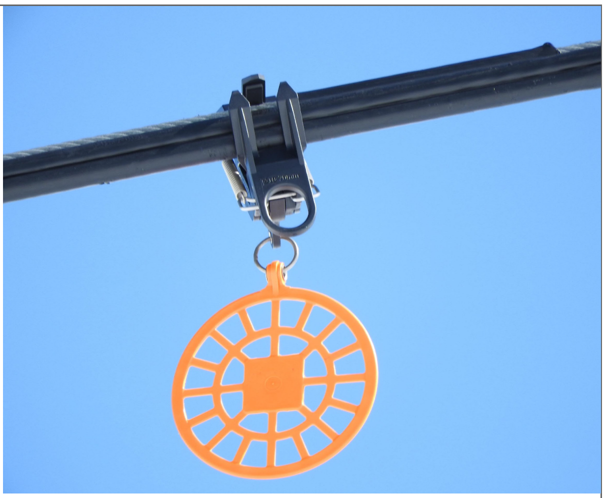
Firefly Ultra Alps<br>Installation on the multipair of chairlifts and gondola<br>Size: 15 cm x 9 cm<br>*(manufacturer: Hammarprodukter)*	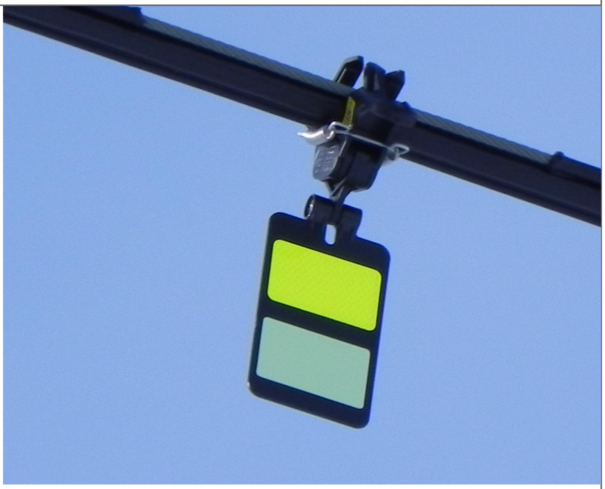
Firefly<br>equipment for power lines<br>Size: 15 cm x10.5 cm<br>*(manufacturer: Hammarprodukter)*	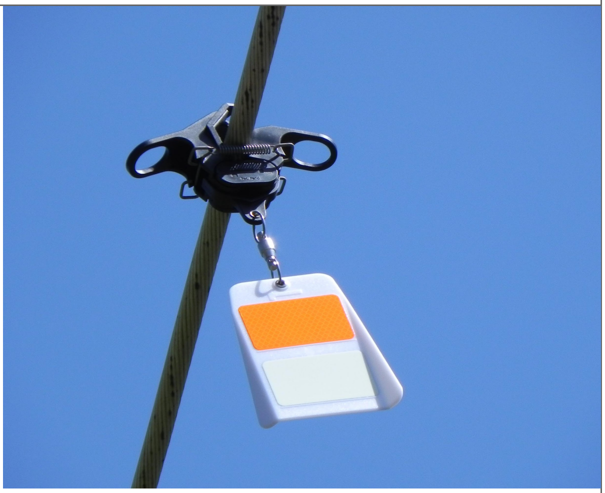

Journal of Experimental Biology • Supplementary information

Table S2. Contrast sensitivity. Steps used to calculate contrast sensitivity, contrast values associated with the vertical sinusoidal achromatic gratings, and the average luminance of the screens.

Step	Michelson Contrast	Inverse Michelson Contrast	Average luminance (cd/m²)
127	1.00	1.00	130.26
120	1.00	1.00	124.79
110	0.99	1.01	116.42
100	0.97	1.03	107.09
90	0.94	1.07	99.86
80	0.90	1.11	93.51
70	0.85	1.18	87.80
60	0.78	1.29	82.73
50	0.69	1.45	77.88
45	0.64	1.57	75.36
40	0.58	1.71	73.18
35	0.52	1.92	71.24
30	0.46	2.17	69.79
29	0.45	2.24	69.33
28	0.43	2.31	68.97
27	0.42	2.39	68.53
26	0.40	2.47	68.28
25	0.39	2.55	67.95
24	0.38	2.65	67.89
23	0.37	2.74	67.31
22	0.35	2.88	67.21
21	0.33	2.99	67.06
20	0.32	3.13	66.67
19	0.30	3.29	66.57
18	0.29	3.45	66.34
17	0.27	3.64	66.09
16	0.26	3.84	65.88
15	0.24	4.10	65.57
14	0.23	4.39	65.33
13	0.21	4.71	65.21
12	0.20	5.07	64.98
11	0.18	5.50	64.71
10	0.17	6.04	64.62
9	0.15	6.62	64.49
8	0.13	7.50	64.13
7	0.12	8.51	63.96
6	0.10	9.62	63.78
5	0.09	11.46	63.57

4	0.07	13.89	63.50
3.5	0.06	16.12	64.05
3	0.05	18.32	63.54
2.5	0.05	21.70	64.04
2	0.04	26.73	63.35
1.5	0.03	35.39	63.89
1	0.02	51.29	63.23
0.5	0.01	82.54	63.76

Table S3. Oligonucleotide primers

ORF primers	Sequence (5' - 3')	ORF size (bp)
s,forward; as, reverse	*nucleotides with underlined capital letters: restriction sites; nucleotides in italics: extra bases for restriction digestion; nucleotides in bold: Kozak sequence*	
Lte-SWS1-ORF-HindIII-s	*ggtgcc*AAGCTT**GCCACC**ATG**G**CCTCGGACGACGACTTCTACCT	1,044
Lte-SWS1-ORF-BsiWI-as	*cggccg*CGTACGAGTGGGGCTGACCTGGCTGG	
Lte-SWS2-ORF-AflII-s	*ggtgcc*CTTAAG**GCCACC**ATG**G**ACCCCCCTCGCCCCC	1,071
Lte-SWS2-ORF-BsiWI-as	*cggccg*CGTACGGGCGGGCGAGACGGAGG	
Lte-Rh2-ORF-HindIII-s	*ggtgcc*AAGCTT**GCCACC**ATG**G**GACAGAAGGTATCAATTTTTA	1,068
Lte-Rh2-ORF-BsiWI-as	*cggccg*CGTACGTGCAGGTGATACTTGGCTGGAGG	
Lte-LW-ORF-HindIII-s	*ggtgcc*AAGCTT**GCCACC**ATG**G**AGGAGTGGGAGGCGGCG	1,089
Lte-LW-ORF-BsiWI-as	*cggccg*CGTACGGGCGGGCGAGACGGAGGA	
**pcDNA5-FLAG_T2A-mruby2 construct verification primers**		
pcDNA5-s	GCTGTTTTGACCTCCATAGAAGA	-
pcDNA5-as	TAGAAGGCACAGTCGAGG	-
pLenti-s	ACCGCATGTTAGCAGACTT	-
mRuby-as	CGGCGGCTTAAACCTTATCGTCG	-

Table S4. Modelling of effective cone sensitivity

Cone type	Opsin λ_{max} (nm)	Droplet type	Droplet cut-off λ_{mid} (nm)	Effective cone λ_{max} (nm)	Upper cone sensitivity (5% threshold) (nm)
SWS1	393 *(a)*	T-type	none	393	491
SWS2	436 *(a)*	C-type	~430 *(b)*	449	534
Rh2	482 *(a)*	Y-type	~523 *(b)*	532	580
LWS	545 *(a)*	R-Type	~586 *(b)*	595	643

(a) this study;
(b) based on MSP values from Hart and Vorobyev 2005 (DOI: 10.1007/s00359-004-0595-3);

Journal of Experimental Biology • Supplementary information
